# Synthesis and Preliminary Immunological Evaluation of a Pseudotetrasaccharide Related to a Repeating Unit of the *Streptococcus pneumoniae* Serotype 6A Capsular Polysaccharide

**DOI:** 10.3389/fmolb.2021.754753

**Published:** 2021-12-13

**Authors:** Elena V. Sukhova, Dmitry V. Yashunsky, Ekaterina A. Kurbatova, Elina A. Akhmatova, Yury E. Tsvetkov, Nikolay E. Nifantiev

**Affiliations:** ^1^ Laboratory of Glycoconjugate Chemistry, N.D. Zelinsky Institute of Organic Chemistry, Russian Academy of Sciences, Moscow, Russia; ^2^ Laboratory of Therapeutic Vaccines, Mechnikov Research Institute for Vaccines and Sera, Moscow, Russia

**Keywords:** *Streptococcus pneumoniae* serotype 6A, capsular polysaccharide, conjugate vaccines, repeating unit, phosphodiester, synthesis

## Abstract

2-Aminoethyl glycoside of the pseudotetrasaccharide α-d-Glc*p*-(1→3)-α-l-Rha*p*-(1→3)-d-Rib-ol-(5-*P-*2)-α-d-Gal*p* corresponding to a repeating unit of the *Streptococcus pneumoniae* type 6A capsular polysaccharide has been synthesized. A suitably protected pseudotrisaccharide α-d-Glc*p*-(1→3)-α-l-Rha*p*-(1→3)-d-Rib-ol with a free 5-OH group in the ribitol moiety and a 2-OH derivative of 2-trifluoroacetamidoethyl α-d-galactopyranoside have been efficiently prepared and then connected *via* a phosphate bridge using the hydrogen phosphonate procedure. Preliminary immunological evaluation of this pseudotetrasaccharide and the previously synthesized pseudotetrasaccharide corresponding to a repeating unit of the capsular polysaccharide of *S. pneumoniae* serotype 6B has shown that they contain epitopes specifically recognized by anti-serogroup 6 antibodies and are able to model well the corresponding capsular polysaccharides. Conjugates of the synthetic pseudotetrasaccharides with bovine serum albumin were shown to be immunogenic in mice.

## Introduction

*Streptococcus pneumoniae* is a clinically important bacterial pathogen that causes serious diseases such as pneumonia, bacteremia, meningitis, otitis media, and others in children and adults (Feikin et al., 2000). More than 90 serotypes of S. *pneumoniae* have been identified according to the chemical structure of their capsular polysaccharides (CPs) (Kamerling, 2000). The CPs are considered to be one of the major factors of bacterial virulence. Of the ∼90 serotypes of *S. pneumoniae*, approximately 20, including the serogroup 6, are responsible for 80–90% of all pneumococcal infections (van Dam et al., 1990). Serogroup 6 belongs to the most frequently revealed pneumococci worldwide (Hausdorff et al., 2000) and in Russian Federation (Sidorenko et al., 2020). Highly immunogenic conjugates of the *S. pneumoniae* serotype 6A CP with carrier proteins were prepared already at an early stage of pneumococcal vaccine development (Chu et al., 1983). The CPs of S. *pneumoniae* serotypes 6A and 6B are the constituents of the modern 13-valent conjugate pneumococcal vaccine (Prevnar13®) licensed for clinical application (Khatun et al., 2017).

However, the use of bacterial CPs for the production of conjugate vaccines has some shortcomings associated with difficulties in the cultivation of bacteria, isolation, purification, and standardization of CPs and uncertainty on conjugation with carrier proteins (Gening et al., 2015; Kaplonek et al., 2018; Gening et al., 2021; Seeberger, 2021). Synthetic regular polysaccharides might be an alternative to CPs; however, their synthesis is complex and time-consuming (Kochetkov et al., 1987). A promising way to conjugate carbohydrate vaccines is based on the use of synthetic oligosaccharides that structurally relate to the CPs and contain epitopes responsible for the induction of protective antibodies. Such oligosaccharides possess a strictly defined chemical structure, do not contain bacterial contaminants, and can be conjugated with carrier proteins in a controlled fashion. Glycoconjugate vaccine candidates containing synthetic oligosaccharide ligands have been actively developed in past decades (Gening et al., 2015; Kaplonek et al., 2018; Micoli et al., 2019; Gening et al., 2021; Javed and Mandal, 2021; Seeberger, 2021; Zhang et al., 2021).

In the framework of our research program aiming at the design of carbohydrate pneumococcal vaccines based on synthetic oligosaccharide ligands structurally related to the CPs, we synthesized a set of oligosaccharides representing fragments of the CPs of serotypes 3 and 14 (Sukhova et al., 2014; Tsvetkov et al., 2017) and investigated their immunological properties (Kurbatova et al., 2013; Akhmatova et al., 2016; Kurbatova et al., 2016; Kurbatova et al., 2017; Kurbatova et al., 2020). Recently, we published the synthesis of spacer-armed pseudotetrasaccharide **1** corresponding to a repeating unit of the *S. pneumoniae* type 6B polysaccharide (Sukhova et al., 2018). In continuation of this program, we describe here the preparation of similar pseudotetrasaccharide **2** that represents the repeating unit of the type 6A CP. Although several oligosaccharides related to the type 6A CP have been synthesized (Slaghek et al., 1991; Parameswar et al., 2007; Parameswar et al., 2008; Parameswar et al., 2009; Chaudhury et al., 2018), none of them contained both the phosphate bridge and a spacer group that enables further conjugation with labels or protein carriers.


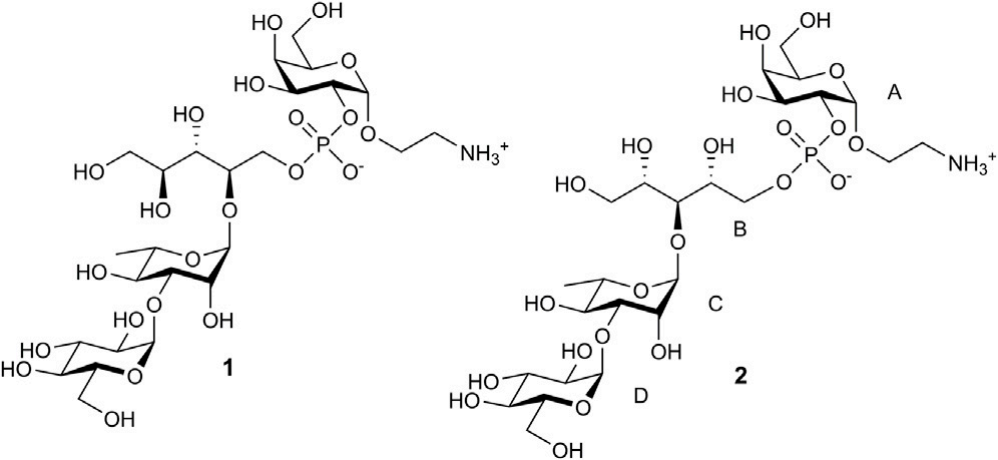


## Materials and Methods

### Chemistry

#### General

All reactions were carried out in solvents purified according to standard procedures. Chemicals were purchased from Acros Organics and Sigma-Aldrich and used without further purification. TLC was performed on Silica Gel 60 F254 plates (Merck Millipore), and visualization was accomplished using UV light or by charring at ∼150°C with 10% (v/v) H_3_PO_4_ in ethanol. Column chromatography was performed on Silica gel 60 (40–63 μm, Merck Millipore). Gel-permeation chromatography of free pseudo-oligosaccharide **2** was carried out on a TSK HW-40(S) column (2.8 × 80 cm) in 0.1-M AcOH using a K-2401 (Knauer) refractometer to monitor the eluate. Biotin conjugates were purified by gel permeation chromatography on a TSK HW-40(S) column (1.6 × 35 cm) in 0.1-M AcOH. Optical rotations were measured using a JASCO P-2000 polarimeter at 18–22°C in solvents specified. Nuclear magnetic resonance (NMR) spectra were recorded on Bruker AMX-400 or Bruker Avance 600 instruments. The spectra of protected carbohydrate derivatives were measured for solutions in CDCl_3_, and ^1^H NMR chemical shifts were referenced to the solvent residual signal (δ_H_ 7.27). ^13^C chemical shifts were referenced to the central resonance of CDCl_3_ (δ_C_ 77.0). ^31^P chemical shifts were measured relatively external 85% H_3_PO_4_. NMR spectra of **2** were measured in D_2_O using acetone (δ_H_ 2.225, δ_C_ 31.45) as the internal standard. The signal assignment was made using COSY and HSQC experiments. Monosaccharide residues in oligosaccharides are denoted upon a description of the NMR spectra as shown in structure **2** and Scheme 4. HRMS (ESI) were obtained on a MicrOTOF II (Bruker Daltonics) instrument. All moisture-sensitive reactions were carried out using dry solvents under dry argon.

#### 2-Chloroethyl 3-*O*-benzyl-α-d-galactopyranoside (8)

Dibutyltin oxide (446 mg, 1.79 mmol) was added to a solution of galactosides **7** (290 mg, 1.19 mmol) in dry MeOH (10 ml), and the mixture was stirred and boiled under reflux for 3 h. The resulting clear solution was concentrated, and the residue was dried in vacuum of an oil pump. The obtained dibutylstannylene derivative was dissolved in dry 1,4-dioxane (8 ml), benzyl bromide (0.85 ml, 7.17 mmol) was added, and the mixture was stirred at 100°C for 5 h. The solvent was evaporated, and the residue was subjected to column chromatography (100:3 chloroform–MeOH) to provide 3-benzyl ether **8** (139 mg, 38%) as an amorphous solid, [α]_D_ + 113 (c 1, CHCl_3_). ^1^H NMR (400 MHz, CDCl_3_): δ_H_ 7.44–7.31 (m, 5H, Ph), 5.03 (d, 1H, *J*_1,2_ = 3.9 Hz, H-1), 4.77, 4.72 (2 d, 2H, *J* = 11.6 Hz, PhC*H*_*2*_), 4.13 (d, 1H, *J*_4,3_ = 3.3 Hz, H-4), 4.06–3.99 (br. m, 1H, H-2), 4.00–3.87 (m, 3H, OC*Ha*CHbCH_2_Cl, H-5, H-6a), 3.84–3.77 (m, 2H, OCHaC*Hb*CH_2_Cl, H-6b), 3.71–3.65 (m, 3H, OCH_2_C*H*_*2*_Cl, H-3), 2.84 (br. s, 1H, OH-4), 2.57 (br. s, 1H, OH-6), 2.31 (d, 1H, *J*_2,OH_ = 8.2 Hz, OH-2). ^13^C NMR (101 MHz, CDCl_3_): δ_C_ 128.6, 128.1, 127.9 (Ar), 99.1 (C-1), 78.2 (C-3), 72.4 (Ph*C*H_2_), 69.9 (C-5), 68.43, 68.37, 68.3 (C-2, C-4, O*C*H_2_CH_2_Cl), 62.9 (C-6), 43.0 (OCH_2_ *C*H_2_Cl). ESIMS: *m*/*z* calcd for C_15_H_21_ClO_6_ [M + Na]^+^ 355.0919. Found: 355.0913.

#### 2-Chloroethyl 3-*O*-benzyl-4,6-*O*-benzylidene-α-d-galactopyranoside (9)

α,α-Dimethoxytoluene (112 μl, 0.75 mmol) and TsOH⋅H_2_O (8 mg, 0.04 mmol) were added to a solution of galactoside **8** (124 mg, 0.37 mmol) in dry CH_3_CN (2 ml); the mixture was heated at 55°C for 5 h, cooled to room temperature and diluted with dichloromethane (30 ml). The solution was washed with aq. saturated NaHCO_3_, dried with Na_2_SO_4_, and concentrated. Column chromatography of the residue (95:5 toluene–acetone) afforded compound **9** (113 mg, 72%) as an amorphous solid, [α]_D_ + 157 (c 1, CHCl_3_). ^1^H NMR (400 MHz, CDCl_3_): δ_H_ 7.63–7.18 (m, 10H, 2 Ph), 5.49 (s, 1H, PhC*H*), 5.11 (d, 1H, *J*_1,2_ = 3.8 Hz, H-1), 4.76 (s, 2H, PhC*H*_*2*_), 4.30–4.23 (m, 3H, H-2, H-4, H-6a), 4.06 (dd, 1H, *J*_6a,6b_ = 12.5 Hz, *J*_5,6b_ = 1.7 Hz, H-6b), 4.00–3.94 (m, 1H, OC*Ha*HbCH_2_Cl), 3.89–3.82 (m, 2H, OCHa*Hb*CH_2_Cl, H-3), 3.85 (br. s, 1H, H-5), 3.73–3.70 (m, 2H, OCH_2_C*H*_2_Cl). ^13^C NMR (101 MHz, CDCl_3_.): δ_C_ 138.7, 138.3, 128.9, 128.4, 128.1, 127.8, 126.2 (Ar), 100.9 (Ph*CH*), 99.4 (C-1), 76.6 (C-3), 73.5 (C-4), 71.5 (Ph*C*H_2_), 69.4 (C-6), 68.6 (O*C*H_2_CH_2_Cl), 67.8 (C-2), 63.3 (C-5), 43.1 (OCH_2_ *C*H_2_Cl). ESIMS: *m*/*z* calcd for C_22_H_25_ClO_6_ [M + Na]^+^ 443.1232. Found: 443.1225.

#### 2-Azidoethyl 3-*O*-benzyl-4,6-*O*-benzylydene-α-d-galactopyranoside (10)

Sodium azide (975 mg, 15 mmol) and 18-crown-6 (34 mg, 0.13 mmol) were added to a solution of galactoside **9** (630 mg, 1.50 mmol) in DMF (15 ml). The mixture was stirred at 60°C for 36 h, cooled to room temperature, diluted with EtOAc (50 ml), and washed with water (3 × 20 ml). The organic solution was dried with Na_2_SO_4_, concentrated, and the residue was chromatographed (20:1 toluene–acetone) to give galactoside **10** (589 mg, 92%) as an amorphous solid, [α]_D_ + 149 (c 1, CHCl_3_). ^1^H NMR (600 MHz, CDCl_3_): δ_H_ 7.57–7.27 (m, 10H, 2 Ph), 5.48 (s, 1H, PhC *H*), 5.10 (d, 1H, *J*_1,2_ = 3.8 Hz, H-1), 4.76 (s, 2H, PhC*H*_*2*_), 4.28 (dd, 1H, *J*_6a,6b_ = 12.4 Hz, *J*_5,6a_ = 2.2 Hz, H-6a), 4.26 (m, 2H, H-4, H-2), 4.06 (dd, 1H, *J*_6a,6b_ = 12.4 Hz, *J*_6b,5_ = 1.8 Hz, H-6b), 4.00–3.95 (ddd, 1H, *J* = 3.2, 8.1, 10.7 Hz, OC*H*aHbCH_2_N_3_), 3.86 (dd, 1H, *J*_3,2_ = 10.1 Hz, *J*_3,4_ = 3.4 Hz, H-3). 3.76–3.69 (m, 2H, OCHa*Hb*CH_2_N_3_, H-5), 3.57 (ddd, 1H, *J* = 3.2, 8.0, 13.3 Hz, 1H, OCH_2_C*Ha*HbN_3_), 3.37 (ddd, 1H, *J* = 3.1, 5.4, 13.3 Hz, OCH_2_CHa*Hb*N_3_), 2.35 (d, 1H, *J*_2,OH_ = 5.8 Hz, OH). ^13^C NMR (151 MHz, CDCl_3_): δ_C_ 138.2, 137.7, 128.8, 128.3, 128.1, 127.8, 126.1 (Ar), 100.8 (Ph*C*H), 99.4 (C-1), 76.3 (C-3), 73.5 (C-4), 71.5 (Ph*C*H_2_), 69.3 (C-6), 67.6 (O*C*H_2_CH_2_N_3_), 67.2 (C-2), 63.2 (C-5), 50.6 (OCH_2_ *C*H_2_N_3_). ESIMS: *m*/*z* calcd for C_22_H_25_N_3_O_6_ [M + Na]^+^ 450.1636. Found: 450.1631.

#### 2-Trifluoroacetamidoethyl 3-*O*-benzyl-4,6-*O*-benzylydene-α-d-galactopyranoside (4)

Triphenylphosphine (613 mg, 2.34 mmol) was added to a solution of galactoside **10** (500 mg, 1.17 mmol) in aq. 90% THF (10 ml), and the mixture was stirred at 60°C for 3 h. The solvent was evaporated, and the residue was dried in vacuum of an oil pump to give crude 2-aminoethyl galactoside. Triethylamine (0.86 ml, 6.18 mmol) and ethyl trifluoroacetate (0.97 ml, 8.13 mmol) were added to a solution of the amine in dry MeOH (10 ml), and the mixture was stirred at room temperature for 2 h and concentrated. The residue was purified by column chromatography (10:1 toluene–acetone) to provide *N*-trifluoroacetyl derivative **4** (442 mg, 76%) as an amorphous solid, [α]_D_ + 128 (c 1, CHCl_3_). ^1^H NMR (400 MHz, CDCl_3_): δ_H_ 8.15 (br. t, 1H, *J* = 5.2 Hz, OCH_2_CH_2_N*H*COCF_3_), 7.58–7.24 (m, 10H, 2 Ph), 5.45 (s, 1H, PhC*H*), 5.01 (d, 1H, *J*_1,2_ = 3.7 Hz, H-1), 4.70, 4.66 (2 d, 2H, *J* = 12.1 Hz, PhC*H*_*2*_), 4.27–4.21 (m, 2H, H-2, H-6a), 4.20 (br. d, 1H, *J*_4,3_ = 3.4 Hz, H-4), 4.01 (dd, 1H, *J*_6b,5_ = 1.8 Hz, *J*_6b,6a_ = 12.6 Hz, H-6b), 3.92 (ddd, 1H, *J* = 3.3, 6.1, 10.8 Hz, OC*Ha*HbCH_2_N), 3.81 (dd, 1H, *J*_3.2_ = 10.0 Hz, *J*_3,4_ = 3.4 Hz, H-3), 3.67 (br. s, 1H, H-5), 3.65–3.55 (m, 2H, OCHa*Hb*CH_2_N, OCH_2_C*Ha*HbN), 3.54–3.45 (m, 1H, OCH_2_CHa*Hb*N), 3.27 (br. s, 1 H, OH). ^13^C NMR (101 MHz, CDCl_3_): δ_C_ 138.1, 137.7, 128.9, 128.5, 128.4, 128.3, 128.09, 127.8, 127.7, 126.1 (Ar), 100.8 (Ph*C*H), 99.9 (C-1), 76.2 (C-3), 73.4 (C-4), 71.3 (Ph*C*H_2_), 69.3 (C-6), 67.9 (C-2), 67.2 (O*C*H_2_CH_2_N), 63.3 (C-5), 39.5 (OCH_2_ *C*H_2_N). ESIMS: *m*/*z* calcd for C_24_H_26_F_3_NO_7_ [M + Na]^+^ 520.1554. Found: 520.1543.

#### 1,4-Di-*O*-benzyl-5-*O*-*tert*-butyldimethylsilyl-2,3-*O*-isopropylidene-d-ribitol (12)

Benzyl bromide (0.61 ml, 5.14 mmol) and 60% suspension of NaH in mineral oil (145 mg, 3.62 mmol) were successively added to a stirred solution of diol **11** (527 mg, 1.72 mmol) in DMF (12 ml) at 0°C. The temperature was gradually increased to 20°C, and stirring was continued for 1 h. The reaction was quenched with MeOH (0.5 ml), and the resulting mixture was distributed between EtOAc (50 ml) and water (30 ml). The organic layer was separated, and the aqueous layer was extracted with EtOAc (2 × 30 ml). The combined organic solutions were washed with water, dried with Na_2_SO_4_, and the solvent was evaporated. The residue was purified by column chromatography (3:1 toluene–EtOAc) to provide compound **12** (703 mg, 84%) as a syrup, [α]_D_ + 23 (c 1, CHCl_3_). ^1^H NMR (600 MHz, CDCl_3_): δ_H_ 7.41–7.24 (m, 10H, 2 Ph), 4.84 (d, 1H, *J* = 11.4 Hz, PhC*Ha*Hb), 4.61 (d, 1H, *J* = 12.4 Hz, PhC*Ha*Hb’), 4.53 (d, 1H, *J* = 12.4 Hz, PhCHa*Hb*’), 4.45–4.42 (m, 1H, H-2), 4.40 (d, 1H, *J* = 11.4 Hz, PhCHa*Hb*), 4.19 (dd, 1H, *J*_3,4_ = 8.8 Hz, *J*_3,2_ = 6.1 Hz, H-3), 4.05 (dd, 1H, *J*_5a,5b_ = 11.2 Hz, *J*_5a,4_ = 2.3 Hz, H-5a), 3.80 (dd, 1H, *J*_5b,5a_ = 11.2 Hz, *J*_5b,4_ = 5.2 Hz, H-5b), 3.75 (dd, 1H, *J*_1a,1b_ = 10.3 Hz, *J*_1a,2_ = 3.1 Hz, H-1a), 3.61 (m, 1H, H-4), 3.54 (dd, 1H, *J*_1b,1a_ = 10.3 Hz, *J*_1b,4_ = 7.8 Hz, H-1b), 1.49 (s, 3H, CH_3_ isopropylidene), 1.39 (s, 3H, CH_3_ isopropylidene), 0.95 (s, 9H, SiC(CH_3_)_3_), 0.10 (s, 6H, Si(CH_3_)_2_). ^13^C NMR (151 MHz, CDCl_3_): δ_C_ 138.4, 138.2, 128.3, 127.8, 127.7, 127.6, 127.5 (Ar), 108.5 (*C*(CH_3_)_2_), 78.3 (C-4), 76.9 (C-2), 75.1 (C-3), 73.4 (Ph*C*H_2_), 71.9 (Ph*C*H_2_), 69.2 (C-1), 63.6 (C-5), 27.9 (CH_3_ isopropylidene), 25.93 (C(*C*H_3_)_3_), 25.48 (CH_3_ isopropylidene), –5.43 (Si(CH_3_)_2_). ESIMS: *m*/*z* calcd for C_28_H_42_O_5_Si [M + Na]^+^ 509.2694. Found: 509.2688.

#### 1,4-Di-*O*-benzyl-d-ribitol (13)

Aq. 40% HF (0.3 ml) was added to a solution of compound **12** (700 mg, 1.44 mmol) in CH_3_CN (6 ml). The mixture was stirred for 30 min at room temperature, diluted with CH_2_Cl_2_ (50 ml), and washed with aq. saturated NaHCO_3_ (2 × 20 ml). The organic solution was concentrated, and the residue was subjected to column chromatography (1:1 toluene–EtOAc → EtOAc) to produce triol **13** (439 mg, 92%) as a syrup, [α]_D_ –28 (c 1, CHCl_3_). ^1^H NMR (600 MHz, CDCl_3_): δ_H_ 7.39–7.27 (m, 10H, 2 Ph), 4.65 (d, *J* = 11.6 Hz, 1H, PhC*Ha*Hb), 4.57–4.50 (m, 3H, PhCHa*Hb*, PhC*H*_2_), 3.99–3.95 (m, 1H, H-2), 3.95–3.91 (m, 1H, H-3), 3.92–3.81 (m, 2H, H-5a,b), 3.65 (dd, *J*_1a,1b_ = 9.8 Hz, *J*_1a,2_ = 3.6 Hz, 1H, H-1a), 3.63–3.59 (m, 2H, H-1b, H-4), 3.37 (d, 1H, *J*_3,OH_ = 5.0 Hz, OH-3), 3.34 (d, 1H, *J*_2,OH_ = 4.2 Hz, OH-2), 2.98 (t, 1H, *J*_5,OH_ = 5.8 Hz, OH-5). ^13^C NMR (151 MHz, CDCl_3_): δ_C_ 137.8, 137.7, 128.5, 128.4, 127.9, 127.8 (Ar), 79.2 (C-4), 73.6 (Ph*C*H_2_), 72.6 (C-3), 71.7 (Ph*C*H_2_), 71.6 (C-1), 70.6 (C-2), 60.8 (C-5). ESIMS: *m*/*z* calcd for C_19_H_24_O_5_ [M + Na]^+^ 355.1516. Found: 355.1514.

#### 1,4-Di-*O*-benzyl-3,5-*O*-benzylidene-d-ribitol (14)

Premixed benzaldehyde (82 μl, 0.81 mmol) and conc. HCl (57 μl, 0.68 mmol) were added to triol **13** (225 mg, 0.68 mmol), and the resulting mixture was stirred for 18 h at room temperature. Dichloromethane (40 ml) was added, and the solution was washed with aq. saturated NaHCO_3_ (2 × 20 ml). The solvent was evaporated, and the residue was chromatographed (3:1 toluene–EtOAc) to produce benzylidene derivative **14** (238 mg, 84%) as a syrup, [α]_D_ –43 (c 1, CHCl_3_). ^1^H NMR (600 MHz, CDCl_3_): δ_H_ 7.47–7.29 (m, 15H, 3 Ph), 5.51 (s, 1H, PhC*H*), 4.63 (d, 1H, *J* = 11.4 Hz, Ph*CHa*Hb), 4.59 (d, 1H, *J* = 11.4 Hz, PhCHa*Hb*), 4.56 (s, 2H, PhC*H*_2_), 4.39 (dd, 1H, *J*_5a,5b_ = 10.8 Hz, *J*_5a,4_ = 4.9 Hz, H-5a), 4.23–4.19 (m, 1H, H-2), 3.92 (dd, 1H, *J*_3,4_ = 9.3 Hz, *J*_3,2_ = 4.1 Hz, H-3), 3.82 (dt, 1H, *J*_4,3_ ∼ *J*_4,5b_ 9.7 Hz, *J*_4,5a_ = 4.9 Hz, H-4), 3.72–3.65 (m, 3H, H-1a, H-1b, H-5b), 2.87 (d, 1H, *J*_OH,2_ = 3.7 Hz, OH-2). ^13^C NMR (151 MHz, CDCl_3_): δ_C_ 137.5, 129.0, 128.6, 128.4, 128.2, 128.1, 128.0, 127.7, 127.6, 126.1 (Ar), 101.0 (Ph*C*H), 80.0 (C-3), 73.4 (Ph*C*H_2_), 72.1 (Ph*C*H_2_), 71.9 (C-2), 70.5 (C-1), 70.2 (C-4), 69.2 (C-5). ESIMS: *m*/*z* calcd for C_26_H_28_O_5_ [M + Na]^+^ 443.1829. Found: 443.1813.

#### 1,2,4-Tri-*O*-benzyl-3,5-*O*-benzylidene-d-ribitol (15)

Benzyl bromide (89 μl, 0.75 mmol) and 60% suspension of NaH in mineral oil (34 mg, 0.85 mmol) were added to a stirred solution of compound **14** (210 mg, 0.50 mmol) in DMF (3 ml) at 0°C. The mixture was allowed to warm to 20°C, and stirring was continued for 3 h. The reaction was quenched with MeOH (0.1 ml), and the resulting mixture was distributed between EtOAc (20 ml) and water (10 ml). The organic layer was separated, and the aqueous layer was extracted with EtOAc (2 × 10 ml). The combined organic solutions were washed with water and dried with Na_2_SO_4_, and the solvent was evaporated. The residue was purified by column chromatography (3:1 toluene–EtOAc) to give compound **15** (224 mg, 88%) as a syrup, [α]_D_ –15 (c 1, CHCl_3_). ^1^H NMR (400 MHz, CDCl_3_): δ_H_ 7.51–7.22 (m, 20H, Ar), 5.48 (s, 1H, PhC*H*), 4.87 (d, 1H, *J* = 11.9 Hz, Ph*Ha*Hb), 4.80 (d, 1H, *J* = 11.9 Hz, PhHa*Hb*), 4.62–4.50 (m, 4H, 2 PhC*H*_*2*_), 4.31 (dd, 1H, *J*_5a,5b_ = 10.7 Hz, *J*_5a,4_ = 5.0 Hz, H-5a), 4.11 (t, 1H, *J* = 6.0 Hz, H-2), 3.99 (br. d, 1H, *J* = 9.5 Hz, H-3), 3.93–3.86 (m, 1H, H-4), 3.85–3.77 (m, 2H, H-1a,b), 3.64 (t, 1H, *J* = 10.3 Hz, H-5b). ^13^C NMR (101 MHz, CDCl_3_): δ_C_ 138.8, 138.4, 137.83, 128.9, 128.4, 128.3, 128.2, 128.1, 127.9, 127.8, 127.6, 127.5, 127.2, 126.2 (Ar), 101.2 (Ph*C*H), 81.3 (C-3), 78.6 (C-2), 73.3, 72.9, 72.4 (3 Ph*C*H_2_), 70.7 (C-1), 69.5 (C-5), 68.8 (C-4). ESIMS: *m*/*z* calcd for C_33_H_34_O_5_ [M + Na]^+^ 533.2298. Found: 533.2281.

#### 1,2,4-Tri-*O*-benzyl-d-ribitol (16)

TsOH⋅H_2_O (112 mg, 0.59 mmol) was added to a solution of benzylidene derivative **15** (370 mg, 0.88 mmol) in 90% aq. CH_3_CN, the mixture was stirred at 70°C for 8 h, then cooled, diluted with chloroform (20 ml), and washed with aq. saturated NaHCO_3_. The aqueous phase was extracted with chloroform (2 × 10 ml), and the combined organic solutions were concentrated. Column chromatography of the residue (3:1 → 7:3 toluene–EtOAc) provided diol **16** (278 mg, 91%) as a syrup, [α]_D_ –15 (c 1, CHCl_3_). ^1^H NMR (400 MHz, CDCl_3_): δ_H_ 7.43–7.18 (m, 15H, Ar), 4.73 (d, 1H, *J* = 11.6 Hz, PhC*Ha*Hb), 4.61 (d, 1H, *J* = 11.6 Hz, PhCHa*Hb*), 4.60 (d, 1H, *J* = 11.5 Hz, PhC*Ha*Hb’), 4.57–4.49 (m, 3H, PhCHa*Hb*’, PhC*H*_*2*_), 4.15–4.10 (m, 1H, H-3), 3.81–3.77 (m, 3H, H-2, H-5a,b), 3.75 (dd, 1H, *J*_1a,1b_ = 10.4 Hz, *J*_1a,2_ = 3.6 Hz, H-1a), 3.65 (dd, 1H, *J*_1b,1a_ = 10.4 Hz, *J*_1b,2_ = 4.8 Hz, H-1b), 3.62 (dt, 1H, *J* = 6.0 Hz, *J* = 4.4 Hz, H-4), 3.01 (d, 1H, *J* = 4.8 Hz, OH-3), 2.50 (br. t, 1H, *J* = 6.1 Hz, OH-5). ^13^C NMR (101 MHz, CDCl_3_): δ_C_ 128.3, 127.8, 127.7, 127.6 (Ar), 78.5 (C-4), 77.7 (C-2), 73.6 (Ph*C*H_2_), 72.2 (Ph*C*H_2_), 72.1 (C-3), 71.7 (Ph*C*H_2_), 69.7 (C-1), 61.3 (C-5). ESIMS: *m*/*z* calcd for C_26_H_30_O_5_ [M + Na]^+^ 445.1985. Found: 445.1975.

#### 1,2,4-Tri-*O*-benzyl-5-*O*-*tert*-butyldimethylsilyl-d-ribitol (6)

Imidazole (62 mg, 0.91 mmol) and *tert*-butyldimethylsilyl chloride (74 mg, 0.50 mmol) were added to a solution of diol **15** (160 mg, 0.38 mmol) in DMF (3 ml), and the mixture was stirred for 24 h at room temperature. The solvent was evaporated, the residue was dissolved in chloroform (30 ml), and the solution was washed with water, dried with Na_2_SO_4_, and concentrated. Column chromatography of the residue (3:1 toluene–EtOAc) gave silyl ether **6** (175 mg, 86%) as a syrup, [α]_D_ –9 (c 1, CHCl_3_). ^1^H NMR (600 MHz, CDCl_3_): δ_H_ 7.42–7.25 (m, 15H, 3 Ph), 4.77 (d, 1H, *J* = 11.7 Hz, PhC*Ha*Hb), 4.74 (d, 1H, *J* = 11.7 Hz, PhC*Ha*Hb’), 4.66 (d, 1H, *J* = 11.7 Hz, PhCHa*Hb*), 4.59 (d, 1H, *J* = 11.7 Hz, PhCHa*Hb*’) 4.58, 4.55 (2 d, 2H, *J* = 12.2 Hz, PhC*H*_*2*_), 4.08 (q, 1H, *J* = 5.5 Hz, H-3), 3.98 (dd, 1H, *J*_5a,5b_ = 11.0 Hz, *J*_5a,4_ = 3.5 Hz, H-5a), 3.89–3.83 (m, 3H, H-2, H-5b, H-1a), 3.73 (dd, 1H, *J*_1b,1a_ = 10.1 Hz, *J*_1b,2_ = 5.0 Hz, H-1b), 3.69 (m, 1H, H-4), 3.21 (d, 1H, *J*_OH,3_ = 5.3 Hz, OH-3), 0.96 (s, 9H, C(CH_3_)_3_), 0.11, 0.10 (2 s, 6H, Si(CH_3_)_2_). ^13^C NMR (151 MHz, CDCl_3_): δ_C_ 138.5, 138.4, 138.1, 128.3, 128.2, 127.8, 127.7, 127.6, 127.5 (Ar), 79.1 (C-4), 78.3 (C-2), 73.5 (Ph*C*H_2_), 72.3, 72.2, 72.1 (2 Ph*C*H_2_, C-3), 70.4 (C-1), 63.9 (C-5), 26.0 (SiC(*C*H_3_)_3_), –5.4 (Si(CH_3_)_2_). ESIMS: *m*/*z* calcd for C_32_H_44_O_5_Si [M + Na]^+^ 559.2850. Found: 559.2839.

#### (6-*O*-Acetyl-2,3,4-tri-*O*-benzyl-α-d-glucopyranosyl)-(1→3)-(2,4-di-*O*-benzoyl-α-l-rhamnopyranosyl)-(1→3)-1,2,4-tri-*O*-benzyl-5-*O*-*tert*-butyldimethylsilyl-d-ribitol (17)

A mixture of thioglycoside **5** (119 mg, 0.13 mmol), acceptor **6** (60 mg, 0.11 mmol), and powdered mol. sieve 4 Å (200 mg) in dichloromethane (3 ml) was stirred at room temperature for 30 min, then cooled to –20°C. NIS (38 mg, 0.17 mmol) was added, stirring was continued for next 10 min, and then, the temperature was decreased to –30°C. TfOH (2 μl, 0.02 mmol) was added, and the resulting mixture was stirred for 45 min, whereas the temperature was gradually increased to –10°C. The reaction was quenched by adding Et_3_N (100 μl), the mixture was diluted with dichloromethane (20 ml), washed with aq. 1 M Na_2_S_2_O_3_ (60 ml) and aq. saturated NaHCO_3_, and concentrated. The residue was purified by column chromatography (4:1 petroleum ether–EtOAc) to produce compound **17** (115 mg, 76%) as a syrup, [α]_D_ + 36 (c 1, CHCl_3_). ^1^H NMR (600 MHz, CDCl_3_): δ_H_ 8.19–6.99 (m, 40H, 8 Ph), 5.60–5.56 (d, 2H, H-2^C^, H-4^C^), 5.30 (s, 1H, H-1^C^), 4.97 (d, 1H, *J*_1,2_ = 3.3 Hz, H-1^D^), 4.76–4.69 (m, 3H, 3 benzylic H), 4.64–4.55 (m, 4H, 4 benzylic H), 4.51 (d, 1H, *J* = 12.1 Hz, benzylic H), 4.47 (d, 2H, *J* = 11.9 Hz, 2 benzylic H), 4.38 (d, 1H, *J* = 12.1 Hz, benzylic H), 4.34–4.27 (m, 4H, H-3^B^_,_ H-3^C^, H-5^C^, benzylic H), 4.06–4.03 (m, 1H, H-2^B^), 3.96 (dd, 1H, *J*_5a,4_ = 3.7 Hz, *J*_5a,5b_ = 11.4 Hz, H-5a^B^), 3.91–3.74 (m, 6H, H-1a,b^B^, H-4^B^, H-5b^B^, H-6a,b^D^), 3.74 (t, 1H, *J* = 9.5 Hz, H-3^D^), 3.71–3.68 (m, 1H, H-5^D^), 3.35 (dd, 1H, *J*_2,3_ = 9.8 Hz, H-2^D^), 1.94 (s, 3H, CH_3_CO), 1.17 (d, 3H, *J*_6,5_ = 6.4 Hz, 3 H-6^C^), 0.90 (s, 9H, SiC(CH_3_)_3_), 0.10, 0.08 (2 s, 6H, Si(CH_3_)_2_). ^13^C NMR (151 MHz, CDCl_3_): δ_C_ 170.5 (CH_3_ *C*O), 166.0, 165.6 (Ph*C*O), 138.5, 138.4, 138.2, 138.0, 133.3, 133.1, 130.1, 129.6, 128.4, 128.3, 128.2, 128.1, 127.8, 127.7, 127.6, 127.4, 127.3 (Ar), 97.2 (C-1^C^), 94.8 (C-1^D^), 81.4 (C-3^D^), 79.2 (C-4^B^), 78.5 (C-2^D^), 77.7 (C-2^B^), 76.5 (C-4^D^), 76.0 (C-3^B^), 75.3, 74.2, 73.1 (3 Ph*C*H_2_), 72.64, 72.56, 72.50 (C-3^C^, C-4^C^, Ph*C*H_2_), 72.3, 71.9 (2 Ph*C*H_2_), 70.3 (C-1^B^), 69.3 (C-2^C^), 69.0 (C-5^D^), 67.1 (C-5^C^), 62.4 (c-6^D^), 62.0 (C-5^B^), 25.9 (SiC(*C*H_3_)_3_), 20.7 (*C*H_3_CO), 17.5 (C-6^C^), –5.35, –5.44 (Si(CH_3_)_2_). ESIMS: *m*/*z* calcd for C_81_H_92_O_17_Si [M + Na]^+^ 1387.5996. Found: 1387.5999.

#### (6-*O*-acetyl-2,3,4-tri-*O*-benzyl-α-d-glucopyranosyl)-(1→3)-(2,4-di-*O*-benzoyl-α-l-rhamnopyranosyl)-(1→3)-1,2,4-tri-*O*-benzyl-d-ribitol (3)

Aq. 40% hydrofluoric acid (100 μl) was added to a solution of **17** (115 mg, 0.09 mmol) in acetonitrile (3 ml). The mixture was stirred for 40 min at room temperature, diluted with dichloromethane (15 ml), and washed with aq. saturated NaHCO_3_ (2 × 20 ml). The organic solution was concentrated, and the residue was purified by column chromatography (1:1 petroleum ether–EtOAc → EtOAc) to afford the title compound (97 mg, 92%) as a colorless syrup, [α]_D_ +46 (c 1, CHCl_3_). ^1^H NMR (400 MHz, CDCl_3_): δ_H_ 8.16–7.01 (m, 40H, 8 Ph), 5.61–5.57 (m, 2H, H-2^C^, H-4^C^), 5.39 (s, 1H, H-1^C^), 4.94 (d, 1H, *J*_1,2_ = 3.5 Hz, H-1^D^), 4.77 (d, 1H, *J* = 11.9 Hz, benzylic H), 4.71 (d, 1H, *J* = 11.9 Hz, benzylic H), 4.66–4.56 (m, 5H, 5 benzylic H), 4.53 (d, 1H, *J* = 12.1 Hz, benzylic H), 4.46 (d, 1H, *J* = 11.2 Hz, benzylic H), 4.44 (d, 1H, *J* = 12.3 Hz, benzylic H), 4.38 (d, 1H, *J* = 12.1 Hz, benzylic H), 4.34 (d, 1H, *J* = 11.2 Hz, benzylic H), 4.32–4.28 (m, 2H, H-3^B^, H-3^C^), 4.21 (dq, 1H, *J*_5,4_ = 9.9 Hz, *J*_5,6_ = 6.2 Hz, H-5^C^), 3.99–3.95 (m, 1H, H-2^B^), 3.93 (dd, 1H, *J*_5a,4_ = 2.6 Hz, *J*_5a,5b_ = 11.9 Hz, H-5a^B^), 3.88–3.74 (m, 6H, H-1a,b^B^, H-4^B^, H-5b^B^, H-6a,b^D^), 3.73 (t, 1H, *J* = 9.5 Hz, H-3^D^), 3.71–3.67 (m, 1H, H-5^D^), 3.35 (dd, 1H, *J*_2,3_ = 9.5 Hz, H-2^D^), 3.33 (t, 1H, *J* = 9.4 Hz, H-4^D^), 1.95 (s, 3H, CH_3_CO), 2.28 (br. s, 1H, OH), 1.21 (d, 3H, *J*_6,5_ = 6.2 Hz, 3 H-6^C^). ^13^C NMR (100 MHz, CDCl_3_): δ_C_ 170.5 (CH_3_ *C*O), 166.2, 165.6 (Ph*C*O), 138.5, 138.1, 138.0, 137.9, 133.3, 133.2, 130.1, 129.6, 128.4, 128.3, 128,2, 128.1, 127.9, 127.7, 127.6, 127.5, 127.4 (Ar), 98.1 (C-1^C^), 95.0 (C-1^D^), 81.4 (C-3^D^), 78.9 (C-4^B^), 78.5 (C-2^D^), 78.0 (C-2^B^), 76.6 (C-3^B^), 76.5 (C-4^D^), 75.3, 74.3, 73.2 (3 Ph*C*H_2_), 72.6 (C-3^C^), 72.5 (C-4^C^), 72.4, 72.3, 71.9 (3 Ph*C*H_2_), 69.7 (C-1^B^), 69.4 (C-2^C^), 69.1 (C-5^D^), 67.4 (C-5^C^), 62.3 (C-6^D^), 60.2 (C-5^B^), 20.7 (*C*H_3_CO), 17.6 (C-6^C^). ESIMS: *m*/*z* calcd for C_75_H_78_O_17_ [M + Na]^+^ 1273.5131. Found: 1273.5115.

#### (2-Trifluoroacetamidoethyl 3-*O*-benzyl-4,6-*O*-benzylidene-α-d-galactopyranoside-2-yl)hydrogenphosphonate Triethylammonium Salt (18)

A mixture of galactoside **4** (98 mg, 0.20 mmol) and phosphorous acid (69 mg, 0.50 mmol) was dried by coevaporation with anhydrous pyridine (3 × 2 ml), and then, pivaloyl chloride (62 μl, 0.5 mmol) was added to a solution of the mixture in anhydrous pyridine (2 ml). The solution was stirred for 6 h at room temperature, and the solvent was evaporated. A solution of the residue in dichloromethane containing 1% (v/v) of Et_3_N (20 ml) was washed with aq. 1-M triethylammonium hydrogencarbonate (2 × 20 ml), dried with Na_2_SO_4_, and concentrated. The residue was purified by column chromatography (40:5:30:12:4:0.1 EtOAc–MeOH–acetone–CH_2_Cl_2_–H_2_O–Et_3_N) to provide compound **18** (101 mg, 92%) as a white amorphous solid, [α]_D_ + 86 (c 1, CHCl_3_). ^1^H NMR (400 MHz, CDCl_3_): δ_H_ 7.54–7.25 (m, 10H, 2 Ph), 6.93 (d, 1H, *J*_P,H_ = 632 Hz, PH), 5.48 (s, 1H, PhC*H*), 5.41 (d, 1H, *J*_1,2_ = 3.8 Hz, H-1), 4.75, 4.69 (2 d, 2H, *J* = 11.8 Hz, PhC*H*_2_), 4.61 (ddd, 1H, *J*_2,3_ = 10.2 Hz, *J*_2,P_ = 8.1 Hz, H-2), 4.27–4.22 (m, 2H, H-4, H-6a), 4.01 (dd, 1H, *J*_6b,5_ = 1.3 Hz, *J*_6b,6a_ = 12.2 Hz, H-6b), 3.94 (dd, 1H, *J*_3,4_ = 3.4 Hz, H-3), 3.91–3.84 (m, 1H, OC*Ha*HbCH_2_NH), 3.78–3.71 (m, 1H, OCHa*Hb*CH_2_NH), 3.68 (br. s, 1H, H-5), 3.56 (br. s, 2H, OCH_2_C*H*_2_NH), 2.77 (q, 6H, *J* = 7.3 Hz, N(C*H*_2_CH_3_)_3_), 1.10 (t, 9H, N(CH_2_C*H*_3_)_3_). ^13^C NMR (100 MHz, CDCl_3_): δ_C_ 138.7, 138.0, 129.7, 128.8, 128.2, 128.0, 127.8, 127,6, 126.2 (Ar), 100.8 (Ph*C*H), 97.4 (C-1), 74.9 (d, *J*_C3,P_ = 7.3 Hz, C-3), 74.5 (C-4), 71.9 (Ph*C*H_2_), 69.3 (C-2, C-6), 65.2 (O*C*H_2_CH_2_N), 62.7 (C-5), 45.4 (N(*C*H_2_CH_3_)_3_), 38.9 (OCH_2_ *C*H_2_N), 8.9 (N(CH_2_ *C*H_3_)_3_). ^31^P NMR (162 MHz, CDCl_3_): δ_P_ 2.92. ESIMS: *m*/*z* calcd for C_24_H_27_F_3_NO_9_P [M + Na]^+^ 584.1268. Found: 584.1251.

#### [(6-*O*-acetyl-2,3,4-tri-*O*-benzyl-α-d-glucopyranosyl)-(1→3)-(2,4-di-*O*-benzoyl-α-l-rhamnopyranosyl)-(1→3)-1,2,4-tri-*O*-benzyl-d-ribitol-5-yl]-(2-trifluoroacetamidoethyl 3-*O*-benzyl-4,6-*O*-benzylidene-α-d-galactopyranoside-2-yl)phosphate Triethylammonium Salt (19)

A mixture of hydrogenphosphonate **18** (32 mg, 48 μmol) and pseudotrisaccharide 3 (70 mg, 56 μmol) was dried by coevaporation with anhydrous pyridine (3 × 2 ml), and then, pivaloyl chloride (28 μl, 0.23 mmol) was added to a solution of the mixture in anhydrous pyridine (1 ml). The solution was stirred for 2 h at room temperature, and then, a freshly prepared solution of iodine (14 mg, 56 μmol) in aq. pyridine (2:1, 0.3 ml) was added. After being stirred for 3 h, the mixture was diluted with dichloromethane, the solution was washed with 1-M aq. Na_2_S_2_O_3_ solution (20 ml), aq. 1-M triethylammonium hydrogencarbonate (2 × 20 ml), dried with Na_2_SO_4_, and concentrated. Column chromatography of the residue (40:5:30:12:4:0.1 EtOAc–MeOH–acetone–CH_2_Cl_2_–H_2_O–Et_3_N) afforded pseudotetrasaccharide **19** (67 mg, 73%) as a white amorphous solid, [α]_D_ +70 (c 1, CHCl_3_). ^1^H NMR (600 MHz, CDCl_3_): δ_H_ 8.06–6.96 (m, 50H, 10 Ph), 5.58 (dd, 1H, *J*_2,1_ = 1.8 Hz, *J*_2,3_ = 3.3 Hz, H-2^C^), 5.54 (t, 1H, *J* = 9.9 Hz, H-4^C^), 5.51 (d, 1H, *J*_1,2_ = 4.0 Hz, H-1^A^), 5.42 (s, 1H, PhC*H*), 5.34 (br. s, 1H, H-1^C^), 4.97 (d, 1H, *J*_1,2_ = 3.5 Hz, H-1^D^), 4.75 (d, 1H, *J* = 11.9 Hz, benzylic H), 4.72 (d, 1H, *J* = 11.7 Hz, benzylic H), 4.69, 4.65 (2 d, 2H, *J* = 11.7 Hz, PhC*H*_2_), 4.62 (d, 1H, *J* = 11.7 Hz, benzylic H), 4.61–4.52 (m, 4H, H-2^A^, 3 benzylic H), 4.47 (d, 1H, *J* = 11.9 Hz, benzylic H), 4.46 (d, 1H, *J* = 12.3 Hz, benzylic H), 4.41 (d, 1H, *J* = 11.0 Hz, benzylic H), 4.37–4.27 (m, 6H, H-3^B^, H-5a^B^, H-3^C^, H-5^C^, 2 benzylic H), 4.19 (d, 1H, *J*_6a,6b_ = 12.3 Hz, H-6a^A^), 4.19–4.15 (m, 1H, H-5b^B^), 4.05 (d, 1H, *J*_4,3_ = 3.8 Hz, H-4^A^), 4.04–4.01 (m, 1H, H-2^B^), 3.96 (q, 1H, *J* = 4.4 Hz, H-4^B^), 3.90 (dd, 1H, *J*_6b,5_ = 1.9 Hz, *J*_6b,6a_ = 12.3 Hz, H-6b^A^), 3.88–3.76 (m, 6H, H-3^A^, H-1a,b^B^, H-6a,b^D^, OC*Ha*HbCH_2_N), 3.74–3.70 (m, 1H, OCHa*Hb*CH_2_N), 3.67 (t, 1H, *J* = 9.2 Hz, H-3^D^), 3.67–3.64 (m, 1H, H-5^D^), 3.62–3.57 (m, 1H, OCH_2_C*Ha*HbN), 3.54–3.48 (m, 1H, OCH_2_CHa*Hb*N), 3.40 (s, 1H, H-5^A^), 3.31 (dd, 1H, *J*_2,3_ = 9.7 Hz, H-2^D^), 3.27 (t, 1H, *J* = 9.4 Hz, H-4^D^), 2.67 (q, 6H, *J* = 7.3 Hz, N(C*H*_2_CH_3_)_3_), 1.92 (s, 3H, CH_3_CO), 1.10 (d, 3H, 3 H-6^C^), 1.02 (t, 9H, N(CH_2_C*H*_3_)_3_). ^13^C NMR (151 MHz, CDCl_3_): δ_C_ 170.5 (CH_3_ *C*O), 165.7, 165.5 (Ph*C*O), 139.0, 138.6. 138.5, 138.2, 138.1, 133.3, 133.0, 130.0, 129,9, 129.6, 129.5, 128.7, 128.4, 128.3, 128.2, 128.0, 127.8, 127.7, 127.6, 127.4, 127.3, 126.2 (Ar), 100.7 (Ph*C*H), 96.4 (C-1^A^), 96.3 (C-1^C^), 94.7 (C-1^D^), 81.3 (C-3^D^), 78.7 (C-2^D^), 78.3 (d, *J*_C4,P_ = 7.7 Hz, H-4^B^), 77.6 (C-2^B^), 76.6 (C-4^D^), 75.5 (C-3^B^), 75.2 (Ph*C*H_2_), 75.0 (d, *J*_C3,P_ = 8.9 Hz, H-3^A^), 74.5 (C-4^A^), 74.2, 73.1 (2 Ph*C*H_2_), 72.6 (C-4^C^), 72.4 (C-3^C^), 72.3, 72.3, 72.0, 71.8 (4 Ph*C*H_2_), 71.1 (d, *J*_C2,P_ = 4.4 Hz, C-2^A^), 70.2 (C-1^B^), 69.3 (2C, C-2^C^, C-6^A^), 69.0 (C-5^D^), 67.2 (C-5^C^), 64.9 (O*C*H_2_CH_2_N), 64.2 (d, *J*_C5,P_ = 4.4 Hz, C-5^B^), 62.4 (2C, C-5^A^, C-6^D^), 45.4 (N(*C*H_2_CH_3_)_3_), 39.0 (OCH_2_ *C*H_2_N), 20.7 (*C*H_3_CO), 17.6 (C-6^C^), 9.1 (N(CH_2_ *C*H_3_)_3_). ^31^P NMR (243 MHz, CDCl_3_): δ_P_ –1.71. ESIMS: *m*/*z* calcd for C_99_H_101_F_3_NO_27_P [M + Na]^+^ 1846.6143. Found: 1846.6131.

#### [α-d-Glucopyranosyl-(1→3)-α-l-Rhamnopyranosyl-(1→3)-d-Ribitol-5-yl]-(2-Aminoethyl α-d-galactopyranoside-2-yl)phosphate (2)

Sodium methoxide (1 M) in MeOH (0.3 ml) was added to a solution of protected pseudotetrasaccharide **19** (38 mg, 21 μmol) in MeOH (3 ml), and the mixture was stirred for 6 h at room temperature. Aq. 1-M NaOH (0.3 ml) was added, the mixture was stirred for 24 h, and then made neutral with 1-M HCl. The solvent was evaporated, and a solution of the residue in water (1 ml) was applied onto a Sep-Pak C-18 cartridge. The cartridge at first was washed with water and then with a gradient of MeOH in water (5 → 80%). Appropriate fractions were pooled and concentrated. The residue was dissolved in an EtOH–EtOAc–water mixture (2:2:1; 2 ml), PdO/C (30 mg) was added, and the resulting mixture was stirred under hydrogen for 8 h at room temperature. The catalyst was filtered off through a Celit layer, washed with aq. 50% MeOH (30 ml), and the combined filtrate and washings were concentrated. The residue was subjected to gel chromatography on a TSK HW-40(S) column in aq. 0.1 M AcOH to produce after freeze-drying free pseudotetrasaccharide **2** (10 mg, 67%) as a white fluffy solid, [α]_D_ + 23 (c 1, water). ^1^H (600 MHz, D_2_O): δ_H_ 5.18 (d, 1H, *J*_1,2_ = 3.8 Hz, H-1^A^), 5.07 (d, 1H, *J*_1,2_ = 3.8 Hz, H-1^D^), 4.99 (br. s, 1H, H-1^C^), 4.27–4.22 (m, 1H, H-2^A^), 4.19 (br. s, 1H, H-2^C^), 4.08–4.04 (m, 2H, H-4^B^, H-5a^B^), 4.04–3.91 (m, 7H, H-3^A^, H-4^A^, H-5^A^, H-2^B^, H-5b^B^, H-5^D^, OC*Ha*HbCH_2_N), 3.83 (dq, 1H, *J*_5,4_ = 9.7 Hz, *J*_5,6_ = 6.5 Hz, H-5^C^), 3.81–3.68 (m, 9H, H-6a,b^A^, H-1a^B^, H-3^B^, H-3^C^, H-3^D^, H-6a,b^D^, OCHa*Hb*CH_2_N), 3.61 (dd, 1H, *J*_1b,2_ = 7.5 Hz, *J*_1b,1a_ = 11.8 Hz, H-1b^B^), 3.56–3.51 (m, 2H, H-4^C^, H-2^D^), 3.43 (t, 1H, *J* = 9.7 Hz, H-4^D^), 3.31–3.20 (m, 2H, OCH_2_C*H*_2_N), 1.27 (d, 3H, *J*_6,5_ = 6.5 Hz, 3 H-6^C^). ^13^C NMR (151 MHz, D_2_O): δ_C_ 101.8 (C-1^C^), 98.4 (C-1^A^), 96.7 (C-1^D^), 80.9 (C-3^B^), 76.6 (C-3^C^), 74.1 (2C, C-2^A^, C-3^D^), 73.1 (C-2^B^), 72.8 (C-5^A^), 72.6 (C-2^D^), 72.4 (C-5^D^), 71.4 (C-4^C^), 70.7, 70.6, 70.5 (4C, C-4^A^, C-4^B^, C-5^C^, C-4^D^), 69.5 (d, *J*_3C,P_ = 5.5 Hz, C-3^A^), 68.2 (C-2^C^), 68.1 (d, *J*_C5,P_ = 5.5 Hz, C-5^B^), 65.0 (O*C*H_2_CH_2_N), 63.8 (C-1^B^), 62.3 (C-6^A^), 61.4 (C-6^D^), 40.3 (OCH_2_ *C*H_2_N), 17.8 (C-6^C^). ^31^P NMR (243 MHz, D_2_O): δ_P_ –0.05. ESIMS: *m*/*z* calcd for C_25_H_48_NO_22_P [M + Na]^+^ 768.2288. Found: 768.2291.

#### Biotin Conjugate (21)

A 0.060-M solution of activated ester **20** in DMF (45.7 µL, 2.7 μmol) and Et_3_N (11 μl, 7.9 μmol) were added to a solution of pseudotetrasaccharide **1** (2.0 mg, 2.3 µmol) in dry DMF (100 µl). The reaction mixture was stirred at ambient temperature for 20 h, and the solvent was removed in vacuum of an oil pump. The residue was subjected to gel-permeation chromatography to produce 2.4 mg (72%) of conjugate **21**, *R*_f_ 0.04 (CH_3_Cl–MeOH–H_2_O, 5:5:1). ^1^H NMR (600 MHz, D_2_O, selected signals) oligosaccharide moiety: δ_H_ 5.08 (d, 1H, *J*_1,2_ = 4.1 Hz, H-1^A^), 5.07 (d, 1H, *J*_1,2_ = 1.5 Hz, H-1^C^), 5.05 (d, 1H, *J*_1,2_ = 3.6 Hz, H-1^D^), 1.24 (d, 3H, *J*_6,5_ = 6.3 Hz, 3 H-6^C^); biotin moiety: δ_H_ 4.53 (dd, 1H, *J*_6a,3a_ = 8.0 Hz, *J*_6a,6_ = 5.0 Hz, H-6a), 4.35 (dd, 1H, *J*_6a,3a_ = 7.9 Hz, *J*_3a,4_ = 4.5 Hz, H-3a), 2.92 (dd, 1H, *J*_6,6'_ = 13.1 Hz, *J*_6a,6_ = 5.0 Hz H-6), 2.71 (d, 1H, *J*_6,6'_ = 13.1 Hz, H-6′), 2.49 (t, 2 H, *J*_3',4'_ = 6.2 Hz, H-4′). ESIMS: *m*/*z* calcd for C_50_H_91_N_4_O_31_PS [M + Na]^+^ 1329. 5018. Found: 1329.4987.

#### Biotin Conjugate (22)

In a similar way, pseudotetrasaccharide **2** was converted into conjugate **22** in 78% yield, *R*_f_ 0.04 (CH_3_Cl–MeOH–H_2_O, 5:5:1). ^1^H NMR (600 MHz, D_2_O, selected signals) oligosaccharide moiety: δ_H_ 5.10 (d, 1H, *J*_1,2_ = 3.9 Hz, H-1^A^), 5.04 (d, 1H, *J*_1,2_ = 3.8 Hz, H-1^D^), 4.95 (br. s, 1H, H-1^C^), 1.24 (d, 3H, *J*_6,5_ = 6.2 Hz, 3 H-6^C^); biotin moiety: δ_H_ 4.54 (dd, 1H, *J*_6a,3a_ = 7.9 Hz, *J*_6a,6_ = 4.8 Hz, H-6a), 4.36 (dd, 1H, *J*_6a,3a_ = 7.9 Hz, *J*_3a,4_ = 4.5 Hz, H-3a), 2.93 (dd, 1H, *J*_6,6'_ = 13.1 Hz, *J*_6a,6_ = 5.0 Hz, H-6), 2.71 (d, 1H, *J*_6,6'_ = 13.0 Hz, H-6′), 2.50 (t, 2H, *J*_3',4'_ = 6.2 Hz, H-4′). ESIMS: *m*/*z* calcd for C_50_H_91_N_4_O_31_PS [M + NH_4_]^+^ 1324.5464. Found: 1324.5440.

### Immunology

#### Bacterial Capsular Polysaccharides

Bacterial CPs used as well coating antigens were obtained from laboratory strains of *S. pneumoniae* serotypes 6A and 6B. The strains were expanded in semisynthetic nutrient media. Isolation of CPs was previously described (Akhmatova et al., 2016). The presence of CP in the preparations was confirmed by NMR spectroscopy.

#### Measurement of the Antibody Level to Pseudotetrasaccharides and Capsular Polysaccharides

Antibody levels were measured by enzyme-linked immunosorbent assay (ELISA) in rabbit sera to *S. pneumoniae* serogroup 6 and *S. pneumoniae* serotype 6B (Statens Serum Institut, Copenhagen, Denmark) obtained from Collective Usage Center of the Mechnikov Research Institute for Vaccine and Sera (Moscow, Russia) or in sera of mice immunized with pseudotetrasaccharide conjugates. The serum obtained upon immunization of mice with the bovine serum albumin (BSA) conjugate of the tetrasaccharide repeating unit of *S. pneumoniae* serotype 14 CP adjuvanted with aluminum hydroxide (Kurbatova et al., 2017) was used as the negative control. OD_450_ values of this immune serum under 1:250 dilution were not lower than 1.5 when tested using the corresponding biotinylated *S. pneumoniae* serotype 14 tetrasaccharide as the well coating material. The results are presented as optical density (OD 450 nm) using 1:200 or 1:250 dilutions of the sera. Antibody levels to biotinylated pseudotetrasaccharides **21** and **22** in the rabbit sera were detected on streptavidin-coated 96-well plates. ELISA assays were performed according to the manufacturer’s instructions (Thermo Fisher Scientific Inc). Briefly, 150-nM solutions of each biotin conjugate diluted in phosphate-buffered saline [PBS (Sigma)] were transferred into streptavidin-coated wells (100 μl/well). Biotin conjugates were incubated for 2 h with shaking (300 rpm) at 22°C. Each well was washed three times with 200 μl of the wash buffer [PBS supplemented with 0.05% Tween 20 (PanReac Applichem, Barcelona, Spain) and 0.1% BSA (Sigma)]. After adding the diluted (1:200) rabbit serum (100 μl), the plates were incubated for 30 min at 22°C. Each well was washed three times with 200 μl of the wash buffer. Secondary goat anti-rabbit or rabbit anti-mouse peroxidase-conjugated IgG (Rockland Immunochemicals, Inc., Pottstown, PA, United States) antibodies (100 μl) were added to each well. After 30 min of incubation with shaking (300 rpm) at 22°C, wells were washed three times with 200 μl of the wash buffer. Enzyme substrate aliquots (100 μl) were added, followed by incubation for 15 min at 22°C. Antibody titers to CPs in rabbit sera were measured using flat-bottom plates (Biochemical LTD, Moscow, Russia) coated with *S. pneumoniae* type 6A or 6B bacterial CP (0.5 μg/well). Optical densities (OD) were determined using an iMark microplate absorbance reader (Bio-Rad, Osaka, Japan) at 450 nm.

#### Antigen-Binding Capacity of CP-Induced Antibodies

To study the antibody-binding capacity in the immune rabbit sera, biotinylated pseudotetrasaccharides **21** or **22** were adsorbed on streptavidin-coated 96-well plates. After adding immune antisera (90 µl/well), a concentration gradient of synthetic pseudotetrasaccharides **1** or **2** or bacterial CPs in PBS (1–10 µg/well) was inoculated (10 µl/well) into the wells. Incubations with ligands and CPs were carried out for 30 min at 20–22°C. Plates were washed three times with 200 µl/well of PBS-Tween 20. Next, working dilutions of peroxidase-conjugated goat anti-rabbit IgG antibodies (Thermo Fisher Scientific) were added, as appropriate. Plates were incubated for 30 min at 22°C and then washed three times with 200 µl/well of PBS-Tween 20. Next, 100 µl/well of TMB was added to stain the bound reaction products. After 15 min, the reactions were quenched with 1 M H_2_SO_4_. ODs were determined at 450 nm with the iMark microplate absorbance reader. The results were presented as 50% inhibitory concentration (IC_50_) values, i.e., the inhibitor concentration that led to a twofold OD decrease and were calculated using calibration curves.

#### Animals

BALB/c male mice (*n* = 18) aged 6–8 weeks were purchased from the Scientific and Production Centre for Biomedical Technologies (Moscow, Russia) and kept in the vivarium of the Mechnikov Research Institute for Vaccines and Sera. The housing, husbandry, blood sampling, and sacrificing conditions conformed to the European Union guidelines for the care and use of laboratory animals. Experimental designs were approved (Protocol # 4, October 2020) by the Mechnikov Research Institute for Vaccines and Sera Ethics Committee.

#### Immunization

Mice (*n* = 6 in each group) were immunized intraperitoneally with pseudotetrasaccharides 6A and 6B conjugated to BSA. The conjugates were obtained as described previously (Sukhova et al., 2014), and adsorbed on aluminum hydroxide (Sigma-Aldrich Co., United States ) served as an adjuvant. Animals were dosed twice, on days 0 and 14 of the experiment. A single dose of the glycoconjugates was 20 µg (carbohydrate content) in saline. Aluminum hydroxide was added in an amount of 250 μg per immunizing dose and stored overnight at 4°C. Non-immunized mice (*n* = 6) served as a control to obtain native serum.

#### Statistical Analysis

Groups were compared using Mann–Whitney rank-sum tests for independent samples. *p*-values ≤0.05 were considered statistically significant using Statistica data analysis software system version 10 (StatSoft Inc., Tulsa, OK, United States).

## Results and Discussion

### Chemistry

**SCHEME 1:**
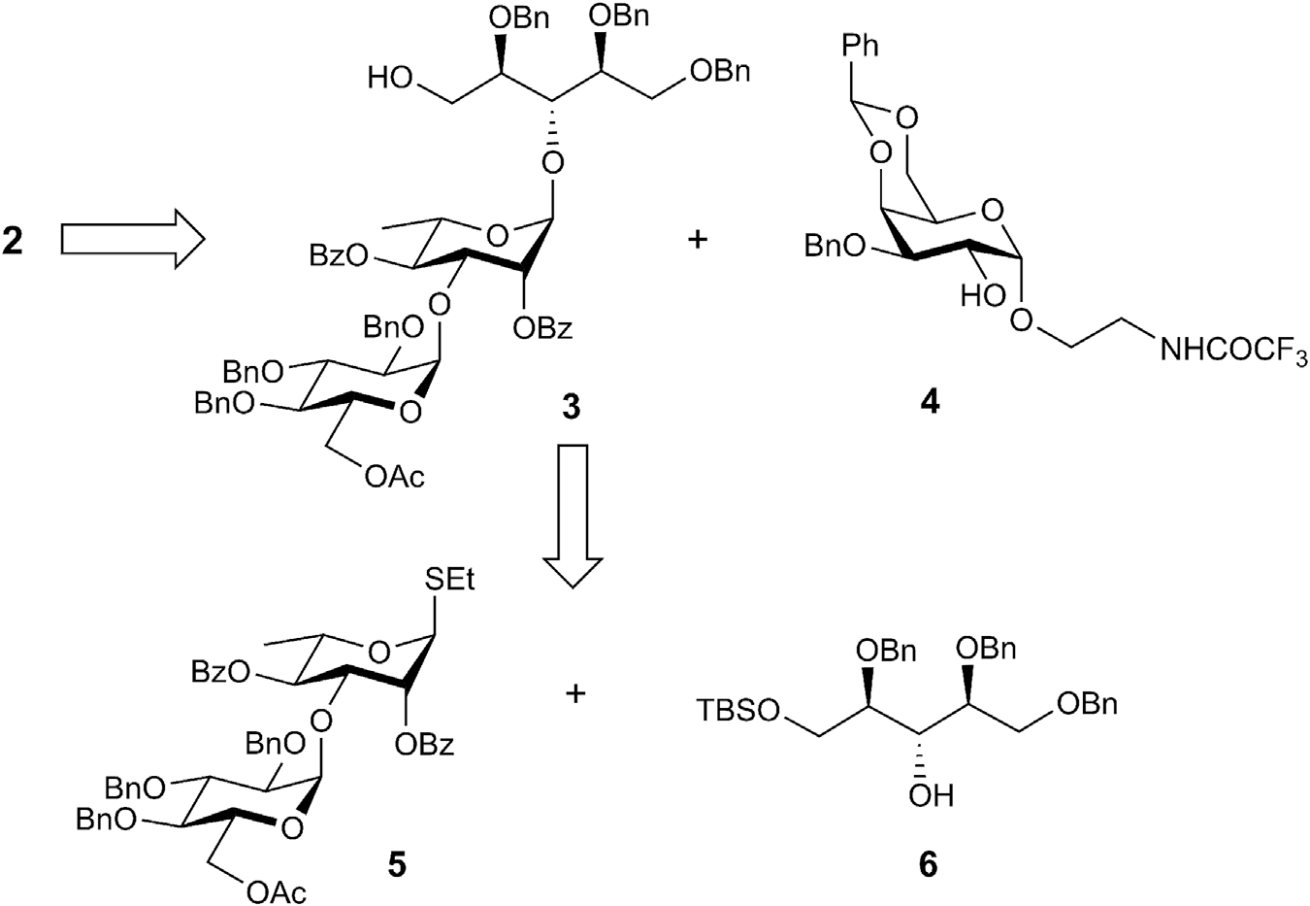
Retrosynthetic analysis of pseudotetrasaccharide **2**.

Pseudotetrasaccharides **1** and **2** possess closely related structures that differ solely in the position of the rhamnosyl-ribitol linkage being (1→3) and (1→4) in the serotypes 6A and 6B, respectively. Accordingly, the synthetic scheme toward **2** was similar to that applied previously to the synthesis of **1** (Sukhova et al., 2018). The target structure **2** was thought to obtain by the connection of pseudotrisaccharide **3** with a free 5-OH group in the ribitol moiety and 2-OH α-galactoside **4** *via* a phosphate bridge (Scheme 1). Compound **3** could be prepared in turn by coupling of properly protected ribitol **6** with known disaccharide thioglycoside **5** (Sukhova et al., 2018).

**SCHEME 2:**
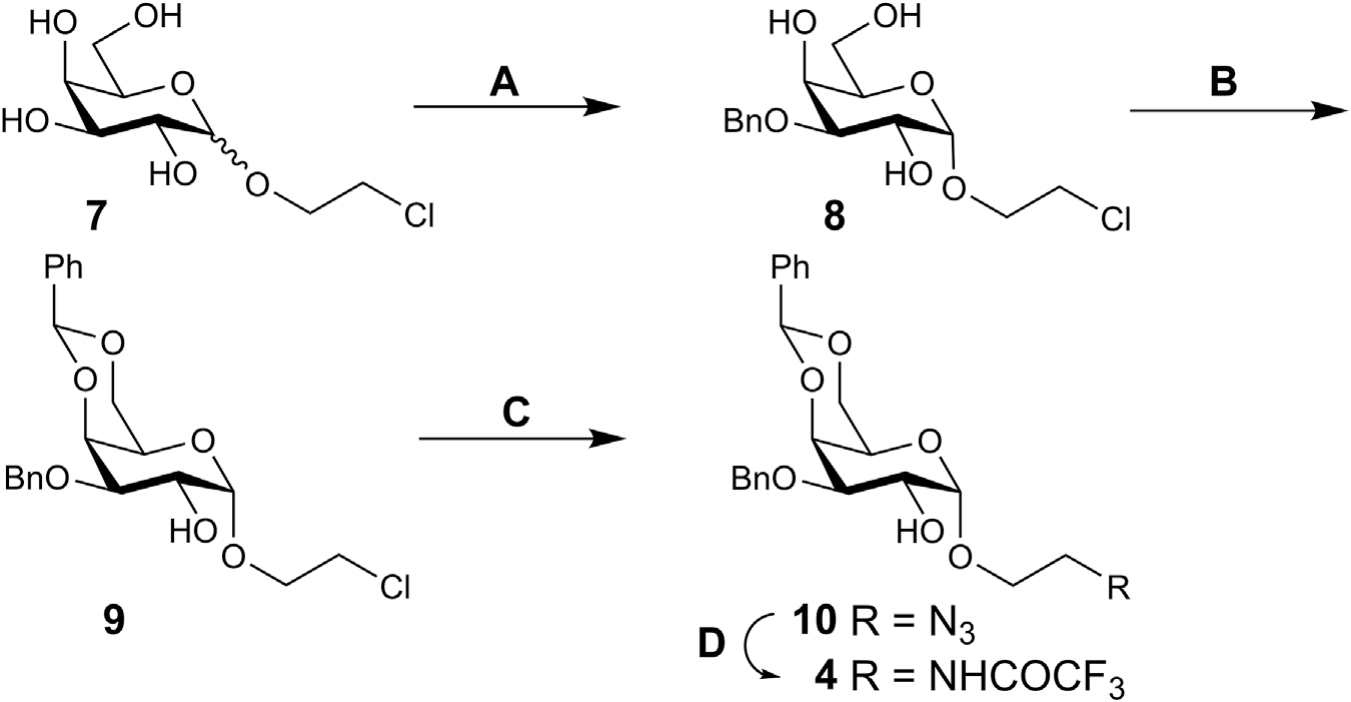
Synthesis of α-galactoside **4**. Reagents and conditions. **(A)** 1. Bu_2_SnO, MeOH, 2. BnBr, 1,4-dioxane, 100°C, 38%; **(B)** PhCH(OMe)_2_, CSA, CH_3_CN, 55°C, 72%; **(C)** NaN_3_, DMF, 60°C, 92%; **(D)** 1. Ph_3_P, THF–water (9:1), 60°C; *2.* CF_3_CO_2_Et, Et_3_N, MeOH, 76% over two steps.

Previously, we applied a benzoyl group for the protection of 3-OH in the α-galactoside (Sukhova et al., 2018). However, migration of the benzoyl group may complicate the phosphorylation step. For this reason, we used benzyl protection at O-3 in the present work. The synthesis of corresponding α-galactoside **4** is outlined in Scheme 2.

An inseparable anomeric mixture of 2-chloroethyl galactosides **7** formed upon Fischer glycosidation of galactose with 2-chloroethanol (Sukhova et al., 2018) was subjected to Bu_2_SnO-mediated benzylation to produce an α,β-mixture of 3-*O*-benzyl ethers, from which pure α-anomer **8** could be isolated by conventional column chromatography in moderate yield. Subsequent reaction of **8** with benzaldehyde dimethyl acetal yielded 4,6-*O*-benzylidene derivative **9**; following substitution of chlorine by azide in the aglycon afforded 2-azidoethyl galactoside **10**. Reduction of the azido group in 10 followed by protection of the amine formed with a trifluoroacetyl group provided requisite 2-OH galactoside **4**.

**SCHEME 3:**
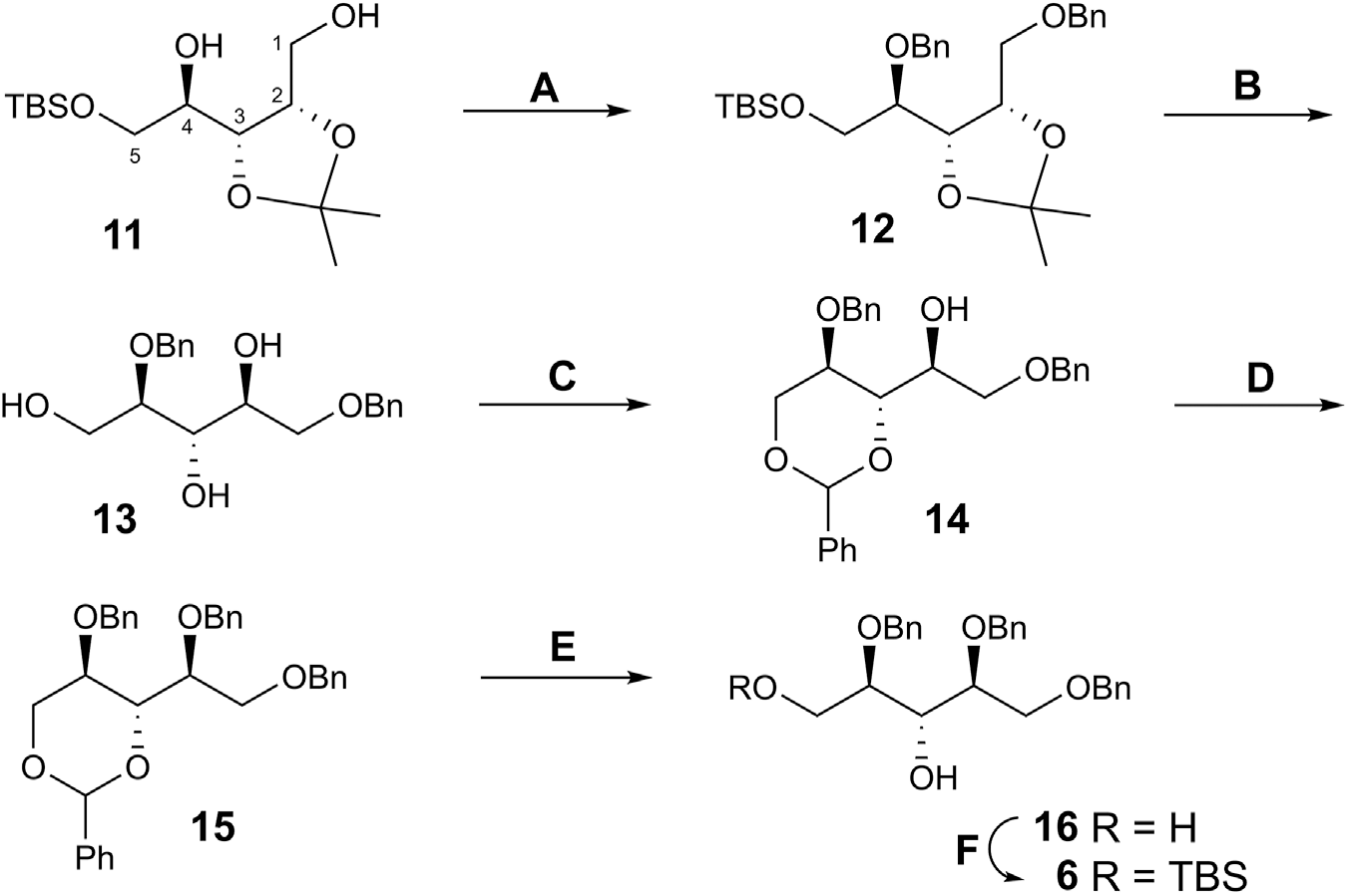
Synthesis of ribitol derivative **6**. Reagents and conditions. **(A)** BnBr, NaH, DMF, 0°C, 84%; **(B)** 40% aq. HF, CH_3_CN, 92%; **(C)** PhCHO, conc. HCl, 84%; **(D)** BnBr, NaH, DMF, 0°C, 88%; **(E)** TsOH⋅H_2_O, 90% aq. CH_3_CN, 70°C, 91%; **(F)** TBSCl, imidazole, DMF, 86%.

A properly protected d-ribitol derivative **6**, in which the hydroxyl groups at C-3 and C-5 could be consecutively glycosylated and phosphorylated, was prepared as follows (Scheme 3). Thus, the benzylation of known isopropylidene acetal **11** (Pan et al., 2005) afforded dibenzyl ether **12**. The synthesis of **12** was described (Pan et al., 2007), but the product was not characterized. Simultaneous removal of the silyl and isopropylidene groups with aq. hydrogen fluoride smoothly produced triol **13**. Treatment of **13** with benzaldehyde in the presence of conc. hydrochloric acid gave 3,5-*O*-benzylidene acetal **14** as the thermodynamically controlled product.

The position of the benzylidene group followed from the strong downfield shift of the signals for C-3 (δ 72.6 → 79.9 ppm) and C-5 (δ 60.8 → 69.1 ppm) in the ^13^C spectrum of **14** compared to triol **13**, whereas the chemical shift of the signal for C-2 changed insignificantly, thus excluding the alternative 2,3-*O*-benzylidene structure. Benzylation of 2-OH in **14** followed by removal of the benzylidene group in product **15** provided 3,5-diol **16**. Selective silylation of the primary OH group in **16** afforded compound **6**. The presence of a correlation peak between H-3 and OH in the COSY spectrum of **6** proved the location of the free hydroxyl group. Thus, requisite glycosyl acceptor **6** has been prepared in six steps from **11** in an overall yield of 46%.

**SCHEME 4:**
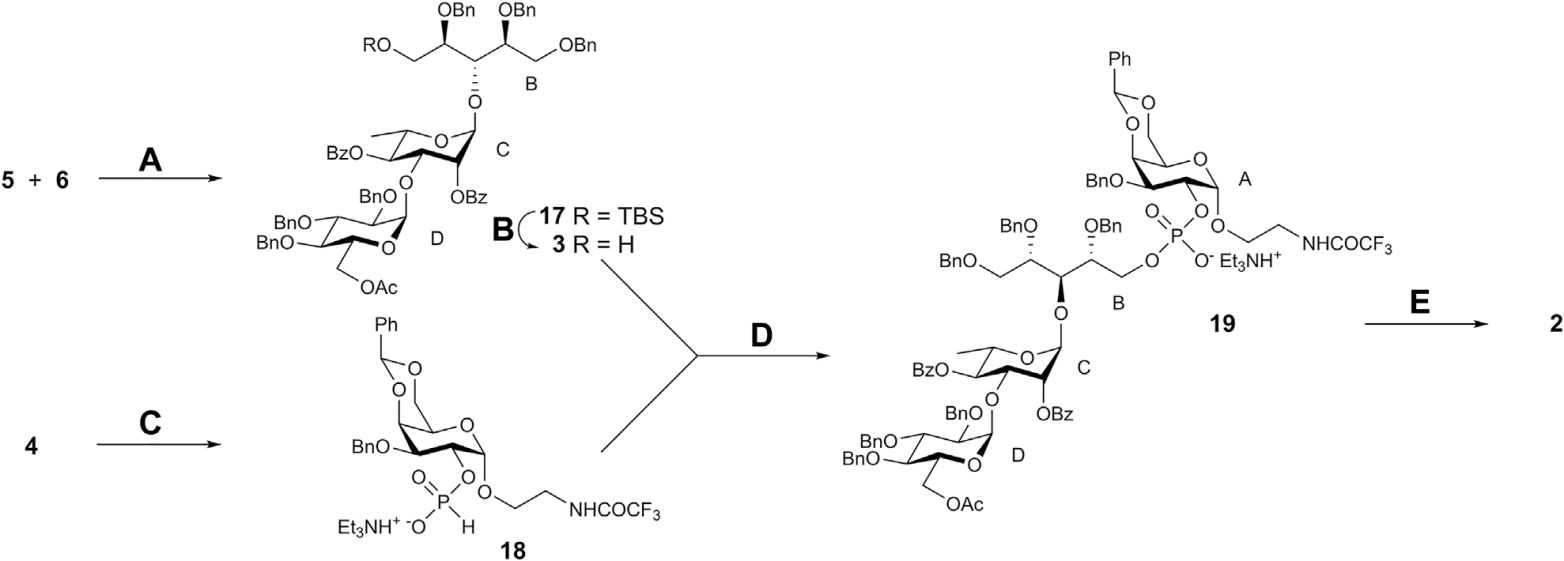
Synthesis of pseudotetrasaccharide **2**. Reagents and conditions. **(A)** NIS, TfOH, MS 4 Å, CH_2_Cl_2_, –25 → –10°C, 76%; **(B)** aq. 40% HF, CH_3_CN, 92%; **(*C*)** H_3_PO_3_, PivCl, pyridine, 92%; **(D)** 1. PivCl, pyridine; 2. I_2_, aq. pyridine (2:1), 73%; **(E)** 1. MeONa, MeOH; 2. NaOH aq. MeOH; 3. H_2_, PdO/C, EtOAc–EtOH–water (2:2:1), 67% over 3 steps.

NIS–TfOH-promoted coupling of thioglycoside **5** with ribitol acceptor **6** afforded pseudotrisaccharide **17** that was desilylated to give derivative **3** suitable for following phosphorylation (Scheme 4). Hydrogenphosphonate (H-phosphonate) procedure (Nikolaev et al., 2007) was applied for the preparation of the phosphodiester from **3** and **4**. Galactoside **4** was converted to H-phosphonate **18** by using phosphorous acid in the presence of pivaloyl chloride (Faurel-Paul et al., 2009). Condensation of **18** with **3** (again in the presence of pivaloyl chloride) followed by oxidation of the intermediate H-phosphonic diester with iodine produced protected phosphodiester **19** in good yield. Total deprotection of **19** included O-deacylation with methanolic MeONa, alkaline hydrolysis of the N-trifluoroacetyl group, and catalytic debenzylation and gave target free phosphodiester **2** in 67% yield. ^1^H and ^13^C NMR data for **2** confirmed the presence of the expected monosaccharide residues. The presence of correlation peaks between ^31^P of the phosphate group and H-2 of galactose and H-5a,b of ribitol in the ^31^P-^1^H HMBC spectrum of **2** proved unequivocally the position of the phosphate bridge.

**SCHEME 5:**
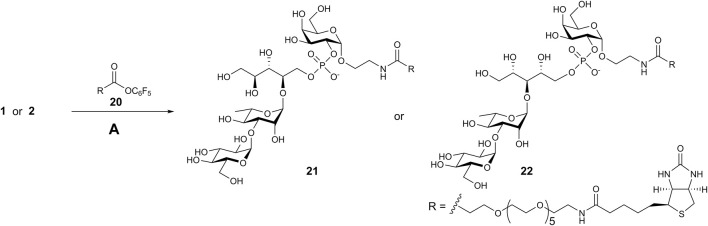
Synthesis of biotin conjugates. Reagents and conditions. **(A)** Et_3_N, DMF, 72% for **21**; 78% for **22**.

Acylation of the spacer amino groups in pseudotetrasacchrides **1** and **2** with biotin-derived pentafluorophenyl ester **20** (Tsvetkov et al., 2012) produced biotin conjugates **21** and **22** (Scheme 5) used as coating antigens upon immunological evaluation of **1** and **2**.

### Immunology

A comparative study of the interaction of the synthetic pseuodotetrasaccharides and CPs of *S. pneumoniae* serotypes 6A and 6B with rabbit immune sera to *S. pneumoniae* serogroup 6 (contains antibodies to serotypes 6A, 6B, and 6C) and *S. pneumoniae* serotype 6B has been carried out to reveal the presence in **1** and **2** of epitopes specifically recognized by anti-serogroup 6 antibodies. Additionally, immunogenicity in mice of pseudotetrasaccharides **1** and **2** conjugated to BSA has been examined.

**FIGURE 1:**
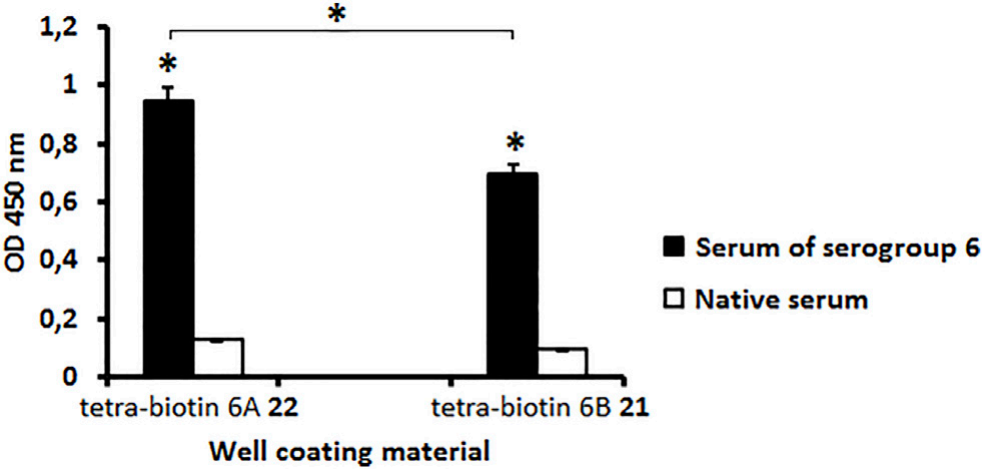
Level of antibodies in pooled rabbit serum to *S. pneumoniae* serogroup 6 determined with biotin conjugates **21** and **22** as well coating materials. Biotinylated pseudotetrasaccharides 6A **22** and 6B **21** were immobilized on surface of streptavidin pre-coated plates. IgG antibody levels are presented as OD_450_. Antibody level to *S. pneumoniae* serogroup 6 was determined in commercial pooled rabbit serum. Pooled native serum obtained from 4 naïve rabbits served as a control (white bars). Each serum was tested four times in 1:200 dilution. Data are displayed as a mean value ± standard deviation (M ± SD). Mann–Whitney Rank Sum tests were used to calculate significance, * *p* < 0.05.

The antibody level in the serum to *S. pneumoniae* serogroup 6 was measured using biotin conjugates **21** and **22** as well coating materials. A native rabbit serum served as a control (Figure 1). Biotinylated pseudotetrasaccharides 6A **22** and 6B **21** revealed a high level of IgG antibodies in the rabbit serum to CPs of *S. pneumoniae* serogroup 6 as compared with the native serum (*P* < 0.05). The level of antibodies recognizing pseudotetrasaccharide 6A **22** was higher than those recognizing pseudotetrasaccharide 6B **21**. It may be a consequence of the higher avidity of antibodies recognizing biotin conjugate **22** or their higher concentration in the pooled serum to *S. pneumoniae* serogroup 6.

**FIGURE 2:**
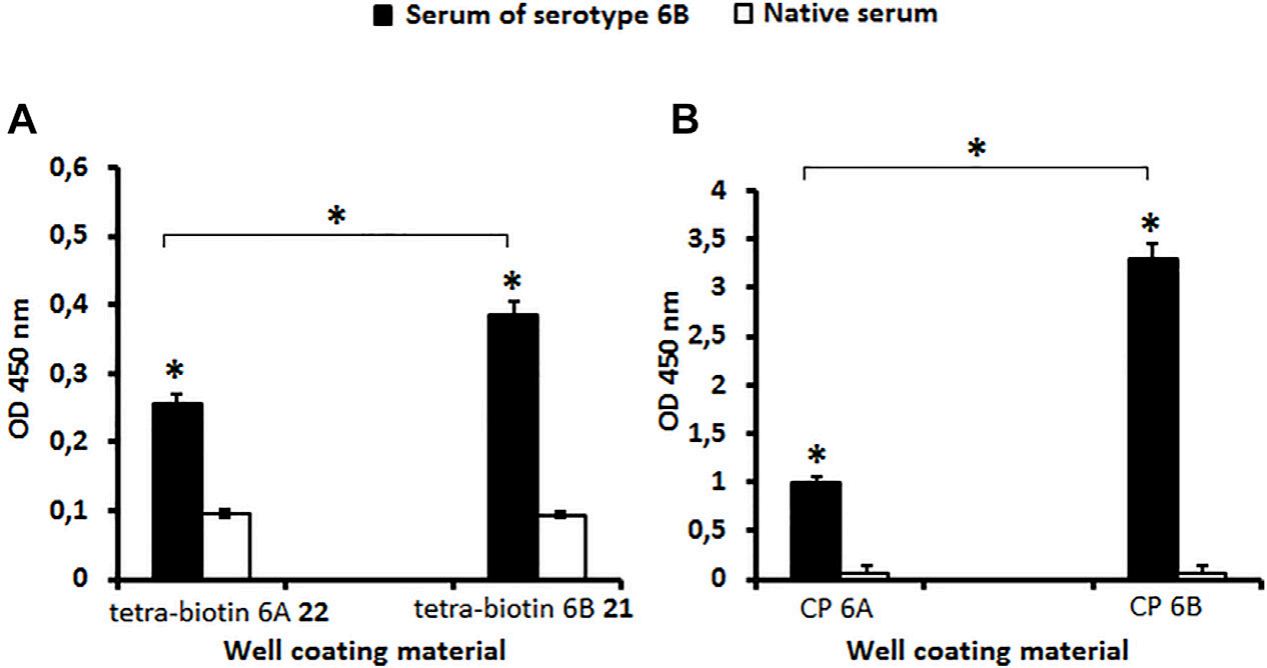
Antibody levels in rabbit serum to *S. pneumoniae* serotype 6B determined with biotin conjugates **21**, **22** **(A)** and CPs 6A, 6B **(B)** as well coating materials. Biotinylated pseudotetrasaccharides 6A **22** and 6B **21** were immobilized on surface of streptavidin pre-coated plates. CPs of *S. pneumoniae* serotypes 6A and 6B were adsorbed on surface of polymeric plates. IgG antibody levels are presented as OD_450_. Antibody level to *S. pneumoniae* serogroup 6B determined in commercial pooled rabbit serum. Pooled native serum obtained from 4 naïve rabbits served as a control (white bars). Each serum was tested four times in 1:250 dilution. Data are displayed as a mean value ± standard deviation. Mann–Whitney Rank Sum tests were used to calculate significance, * *p* < 0.05.

Antibodies in the serum to *S. pneumoniae* serotype 6B possessed the capacity to bind to biotinylated tetrasaccharides **21** and **22** (Figure 2A) as well as to CPs 6A and 6B (Figure 2B) as compared with the native serum (*P* < 0.05). The level of IgG antibodies in the serum to CP *S. pneumoniae* serotype 6B was higher than in the native serum (*P* < 0.05) measured using biotinylated pseudotetrasaccharide **21** and CP 6B (Figures 2A,B). The level of antibodies revealed in the systems biotinylated pseudotetrasaccharide 6B **21** or CP 6B/serum 6B was higher than in the systems biotinylated pseudotetrasaccharide 6A **22** or CP 6A/serum 6B (*P* < 0.05). Biotinylated pseudotetrasaccharide 6A **22** and CP 6A used as well coating antigens revealed a cross-reaction with IgG antibodies in the serum to *S. pneumoniae* serotype 6B, thus confirming the presence of common epitopes in 6A and 6B CPs.

**FIGURE 3:**
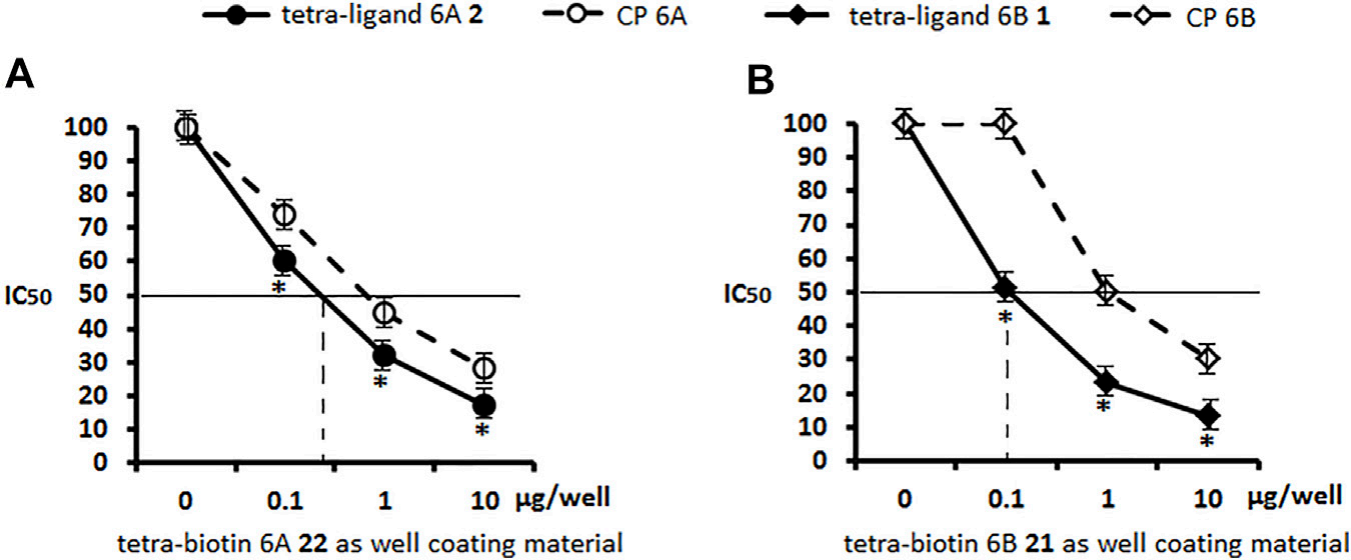
Inhibition of antibodies in sera to *S. pneumoniae* serogroup 6 with pseudotetrasaccharide ligands **2** **(A)** and **1** **(B)** compared to CPs 6A and 6B using biotin conjugates 22 and 21 as well coating materials. Biotinylated pseudotetrasaccharides 6A **22** and 6B **21** were immobilized on surface of streptavidin pre-coated plates. After adding ligands or CPs to serum, OD_450_ values corresponding to IgG antibody level in commercial pooled rabbit serum to *S. pneumoniae* serogroup 6 in 1:200 dilution were determined. Each sample was tested four times. Lines intersecting curves demonstrate 50% inhibitory concentrations that led to a twofold OD decrease. Data are presented as a mean value of optical density ±standard deviation. Significance of difference between pseudotetrasaccharide ligands 6A **2**, 6B **1**, and CPs 6A, 6B was calculated using OD_450_ at each point, Mann–Whitney Rank Sum test, * *p* < 0.05.

The ability of pseudotetrasaccharide ligands **1**, **2** and CPs 6A, 6B to inhibit binding of antibodies in sera to *S. pneumoniae* serogroup 6 or serotype 6B to well coating antigens **21** and **22** was studied. Ligand 6A **2** blocked interaction of antibodies in the serogroup 6 serum with biotinylated pseudotetrasaccharide 6A **22** adsorbed on a streptavidin-coated plate (Figure 3A). Similarly, ligand 6B **1** exhibited a high ability to inhibit binding between the serogroup 6 serum and biotinylated pseudotetrasaccharide 6B **21** (Figure 3B). In both cases, ligands **1** and **2** demonstrated at all concentrations higher antibody binding capacity in the serogroup 6 immune serum than CPs 6A and 6B. The obtained data confirmed a higher inhibitory activity of the pseudotetrasaccharide ligand 6B **1** in the homologs system biotinylated pseudotetrasaccharide 6B **21**/serum 6B (Figure 3B) as compared with the pseudotetrasacchride ligand 6A **2** in the heterologous system biotinylated pseudotetrasaccharide 6A **22**/serum 6B (Figure 3A) with IC_50_ values 0.5 and 0.9 μg/ml respectively.

**FIGURE 4:**
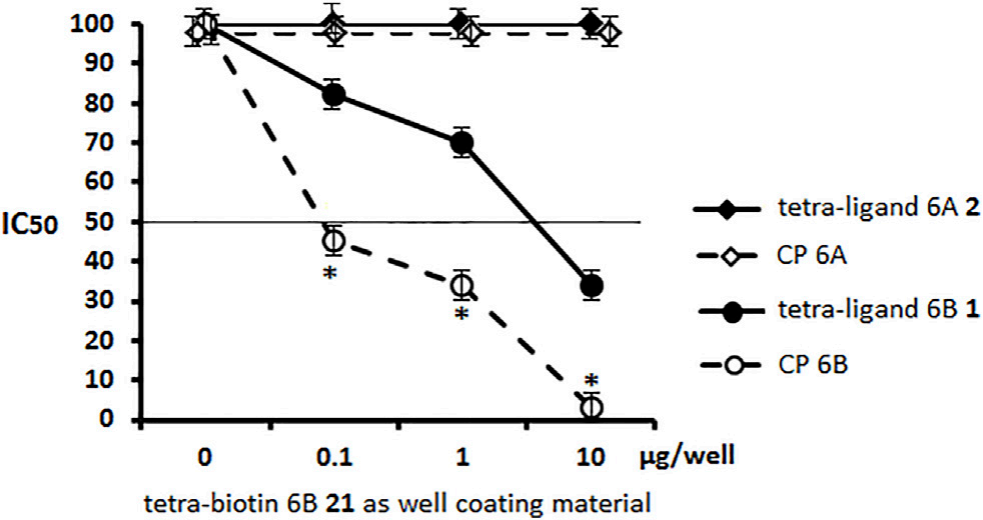
Inhibitory activity of pseudotetrasaccharide ligands **1**, **2** and CPs 6A, 6B in sera to *S. pneumoniae* serotype 6B using biotin conjugate **21** as well coating material Biotinylated pseudotetrasaccharide 6B **21** was immobilized on surface of streptavidin pre-coated plates. After adding ligands 6A **2**, 6B **1** or CPs 6A, 6B, OD_450_ values corresponding to IgG antibody levels in rabbit serum to *S. pneumoniae* serotype 6B in 1:200 dilution were determined. Each sample was tested four times. Line intersecting curves demonstrates 50% inhibitory concentration that led to a two-fold OD decrease. Data presented as a mean value of optical density ± standard deviation. Significance of difference between CP 6B and other samples was calculated using OD_450_ at each point, Mann–Whitney Rank Sum test, * *p* < 0.05.

Pseudotetrasaccharide ligand 6A **2** and CP 6A displayed no inhibitory capacity in the serum to *S. pneumoniae* serotype 6B. Unlike ligand **2**, ligand 6B **1** was able to block the binding of antibodies at a rather high concentration of 8–10 µg/well, being inferior in this respect to CP 6B (Figure 4). The obtained results demonstrated the specificity of antibody recognition using biotin conjugate **21** as well coating material and high specificity of the ELISA inhibition assay to reveal minor structural differences between pseudotetrasaccharide ligands 6A **2** and 6B **1**.

**FIGURE 5:**
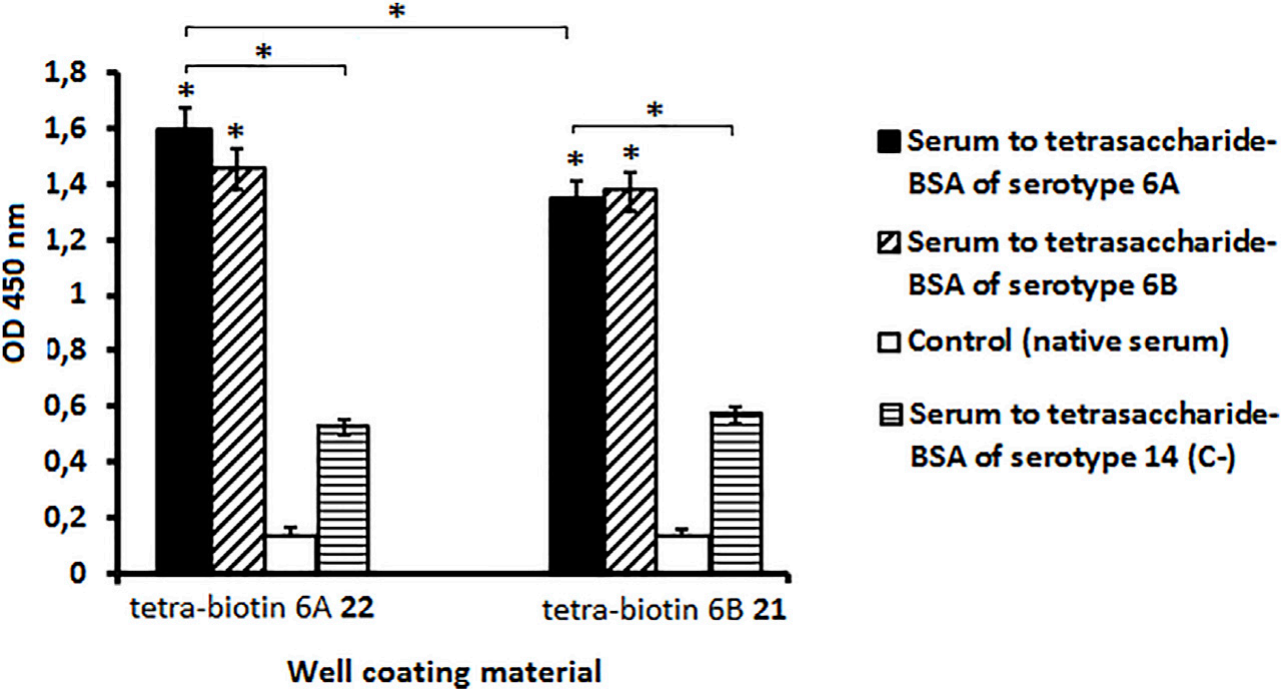
Levels of IgG antibodies elicited by BSA conjugates of pseudotetrasaccharides **1** and **2**. BALB/c mice (*n* = 6 for each conjugate) received two intraperitoneal immunizations with conjugated pseudotetrasaccharides 6A **2** and 6B **1** adjuvanted with aluminum hydroxide. Biotinylated pseudotetrasaccharides 6A **22** and 6B **21** were immobilized on surface of streptavidin pre-coated plates. Antibody levels to conjugates were evaluated in ELISA. Serum obtained from mice immunized with BSA conjugate of tetrasaccharide *S. pneumoniae* serotype 14 adjuvanted with aluminum hydroxide served as negative control (C-). Pooled serum samples were tested six times in 1:250 dilution. Data are displayed as a mean value ± standard deviation. Mann–Whitney Rank Sum tests were used to calculate significance, * *p* < 0.05.

The immunogenicity in mice of pseudotetrasaccarides 6A **2** and 6B **1** conjugated to BSA was also studied (Figure 5). The level of IgG antibodies to both glycoconjugates exceeded the level of antibodies in intact mice (*P* < 0.05). IgG-antibodies induced by the BSA conjugate of the *S. pneumoniae* serotype 14 tetrasaccharide (Kurbatova et al., 2017) (negative control) possessed a low binding capacity for biotinylated pseudotetrasaccharides **21** and **22** compared with antibodies in the sera to pseudotetrasaccharide–BSA conjugates of serotypes 6A and 6B (*P* < 0.05). This result demonstrated the carbohydrate specificity of the antibodies elicited by the BSA conjugates of pseudotetrasaccharides **1** and **2**. There were no differences in the level of antibodies induced to BSA conjugates of pseudotetrasaccharides 6A **2** and 6B **1** when biotin conjugates **22** and **21**, respectively, were used as well coating antigens. Biotinylated pseudotetrasaccharides 6A **22** and 6B **21** immobilized on streptavidin pre-coated plates cross-reacted with IgG antibodies induced by the BSA conjugates of pseudotetrasaccharides 6A **2** and 6B **1**. The level of antibodies induced to the conjugated pseudotetrasaccharide 6A **2** tested on biotinylated pseudotetrasaccharide 6A **22** was higher than that on biotinylated pseudotetrasaccharide 6B **21** (*P* < 0.05). This may be a consequence of small differences in the structure of pseudotetrasaccharides 6A and 6B. Cross-reactions of antibodies to conjugated pseudotetrasaccharides 6A **2** and 6B **1** demonstrate the presence of a common antigenic epitope(s).

The immunological study of pseudotetrasaccharides **1** and **2** related to CPs of *S. pneumoniae* serotypes 6A and 6B revealed slight antigenic differences between them. Anti-CP antibodies in the serum to *S. pneumoniae* serotype 6B cross-reacted with biotinylated pseudotetrasaccharide 6A **22** in ELISA. Pseudotetrasaccharides **1** and **2** conjugated to BSA were shown to be immunogenic in mice. Antibodies induced by those conjugates cross-reacted with biotinylated pseudotetrasaccharides 6A **22** and 6B **21**. These results indicated the presence of a common epitope in CPs of *S. pneumoniae* serotypes 6A and 6B.

## Conclusion

To summarize, we have efficiently synthesized the spacer-armed pseudotetrasacchride corresponding to a repeating unit of the CP of *S. pneumoniae* serotype 6A. The hydrogen phosphonate procedure was applied at the key step of connecting the monosaccharide and pseudotrisaccharide blocks *via* a phosphate bridge. Preliminary immunological evaluation has shown that synthetic pseudotetrasaccharides **1** and **2** contain epitopes specifically recognized by anti-serogroup 6 antibodies and are able to model well the corresponding CPs. Conjugates of pseudotetrasaccharides **1** and **2** with BSA were shown to be immunogenic in mice. Detailed examination of immunogenicity of those conjugates, opsonophagocytic activity of conjugate-induced sera, and protective activity is now in progress and will be reported in due course.

## Data Availability

The original contributions presented in the study are included in the article/Supplementary Material; further inquiries can be directed to the corresponding author.
