# Supplementary material for: Synthesis and Preliminary Immunological Evaluation of a Pseudotetrasaccharide Related to a Repeating Unit of the Streptococcus pneumoniae Serotype 6A Capsular Polysaccharide
Source: Front Mol Biosci. 2021 Dec 13;8:754753. doi: 10.3389/fmolb.2021.754753 (PMC8710661; doi:10.3389/fmolb.2021.754753)

Synthesis and immunological evaluation of a pseudotetrasaccharide related to a repeating unit of the *Streptococcus pneumoniae* serotype 6A capsular polysaccharide

Elena V. Sukhova^1^, Dmitry V. Yashunsky^1^, Ekaterina A. Kurbatova^2^, Elina A. Akhmatova^2^, Yury E Tsvetkov^1^, Nikolay E. Nifantiev^1^*

^1^Laboratory of Glycoconjugate Chemistry, N.D. Zelinsky Institute of Organic Chemistry, Russian Academy of Sciences, Moscow, Russian Federation

^2^Laboratory of Therapeutic Vaccines, Mechnikov Research Institute for Vaccines and Sera, Moscow, Russian Federation

Supplementary Material

**^1^H and ^13^C NMR spectra of synthesized compounds**


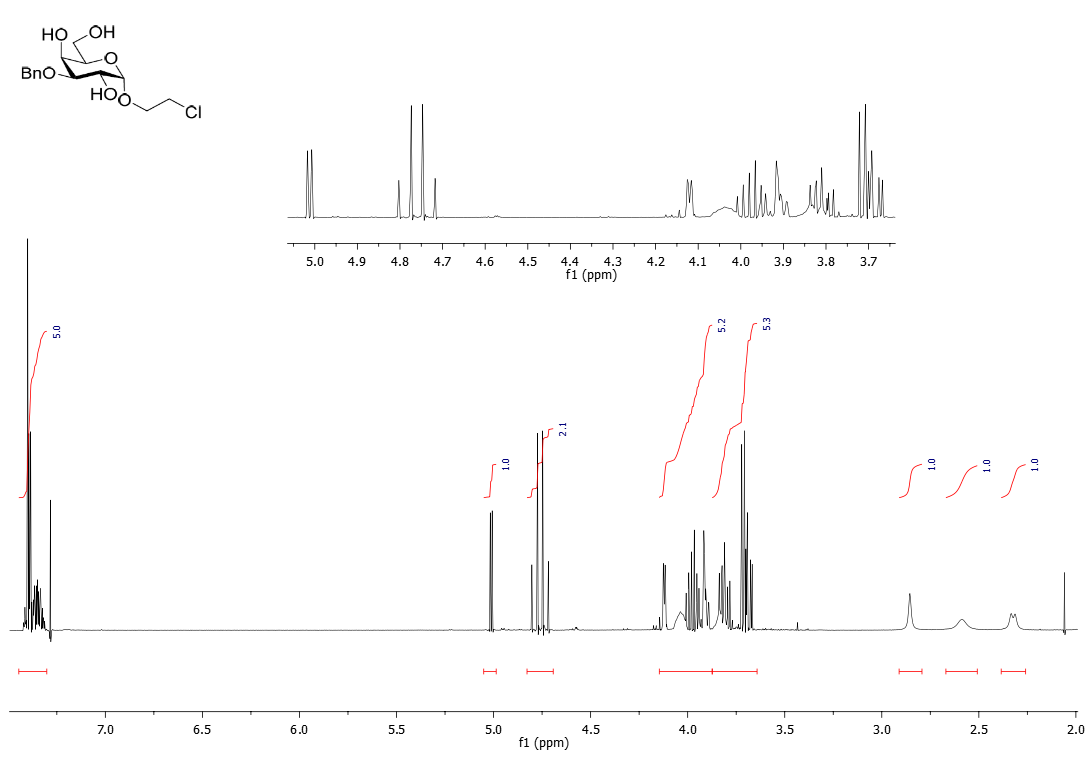


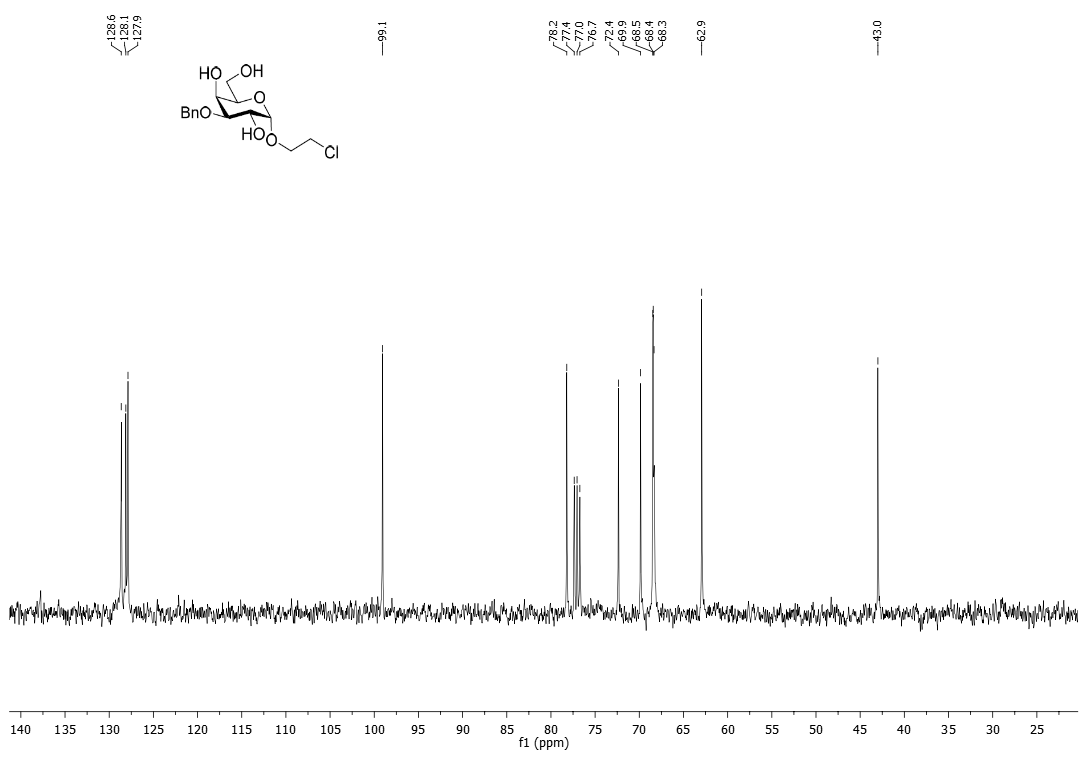


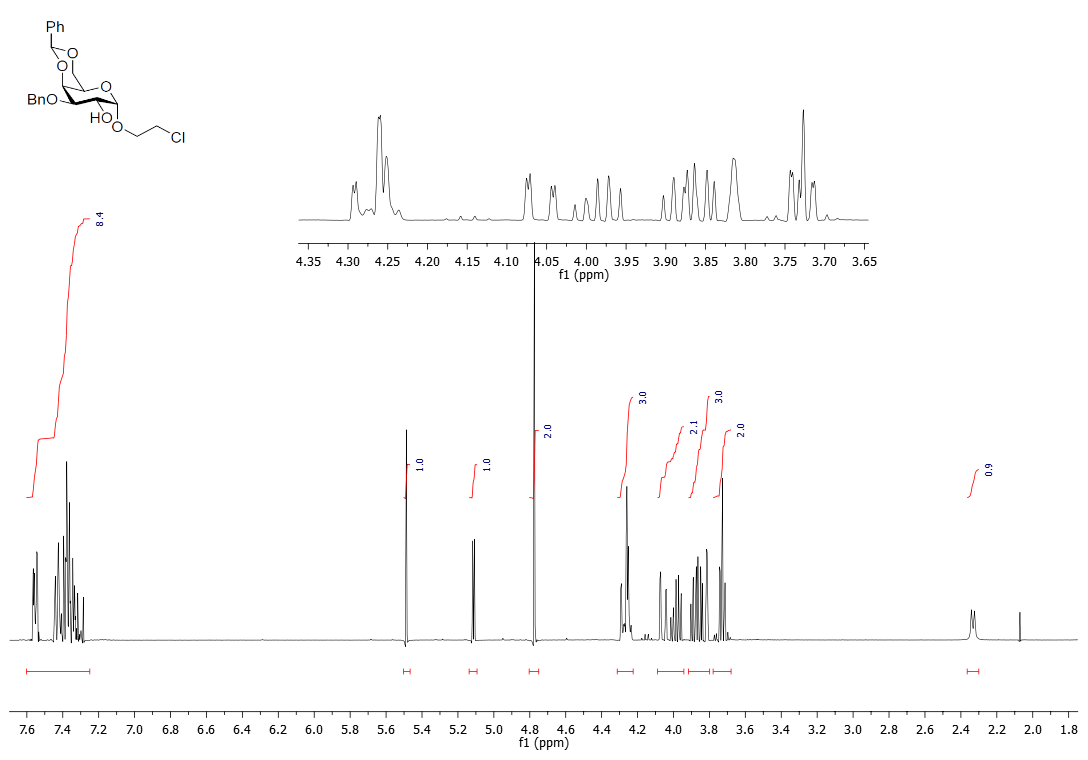


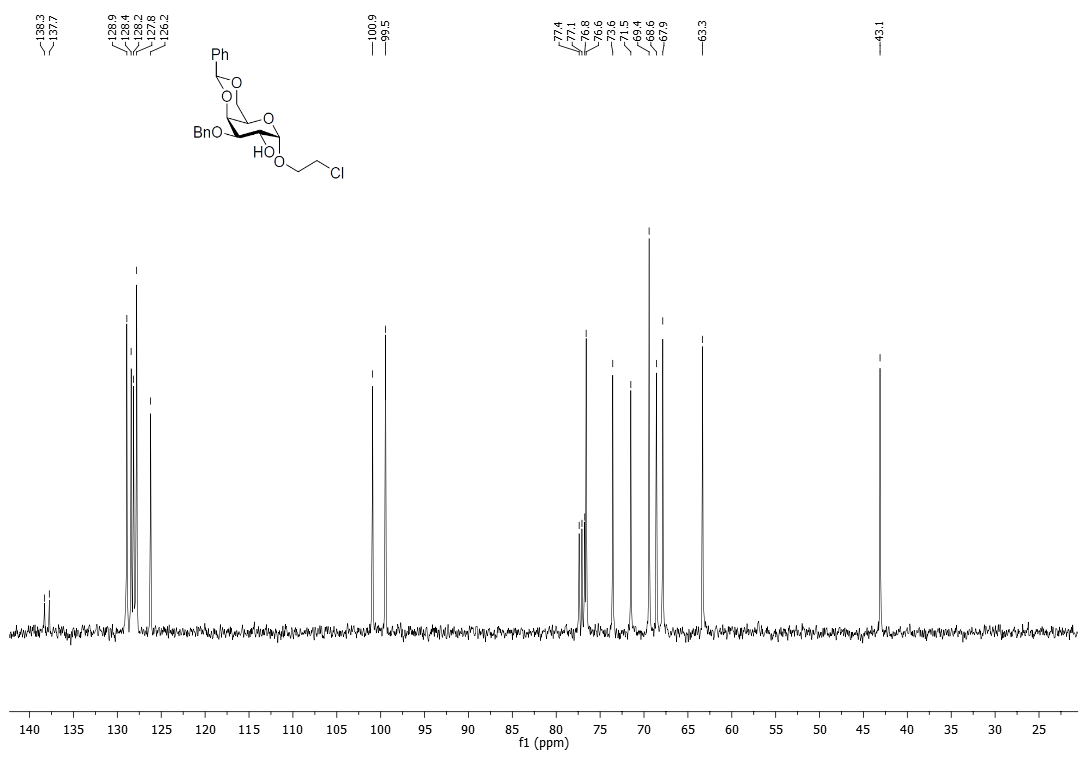


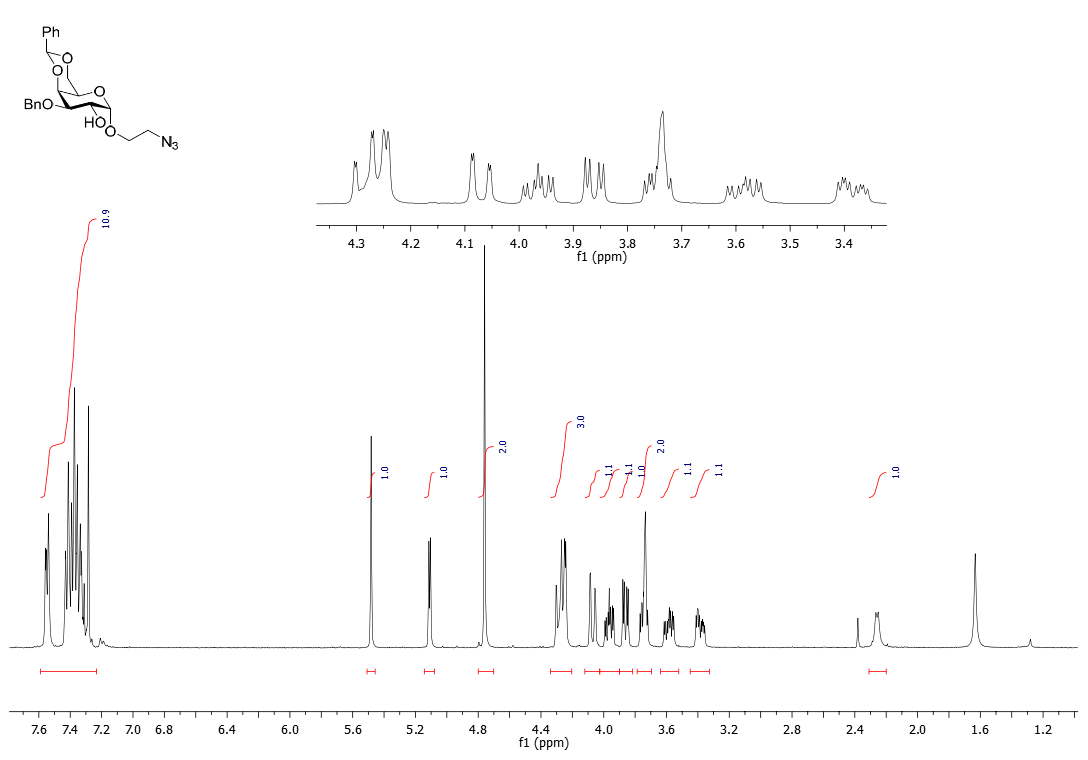


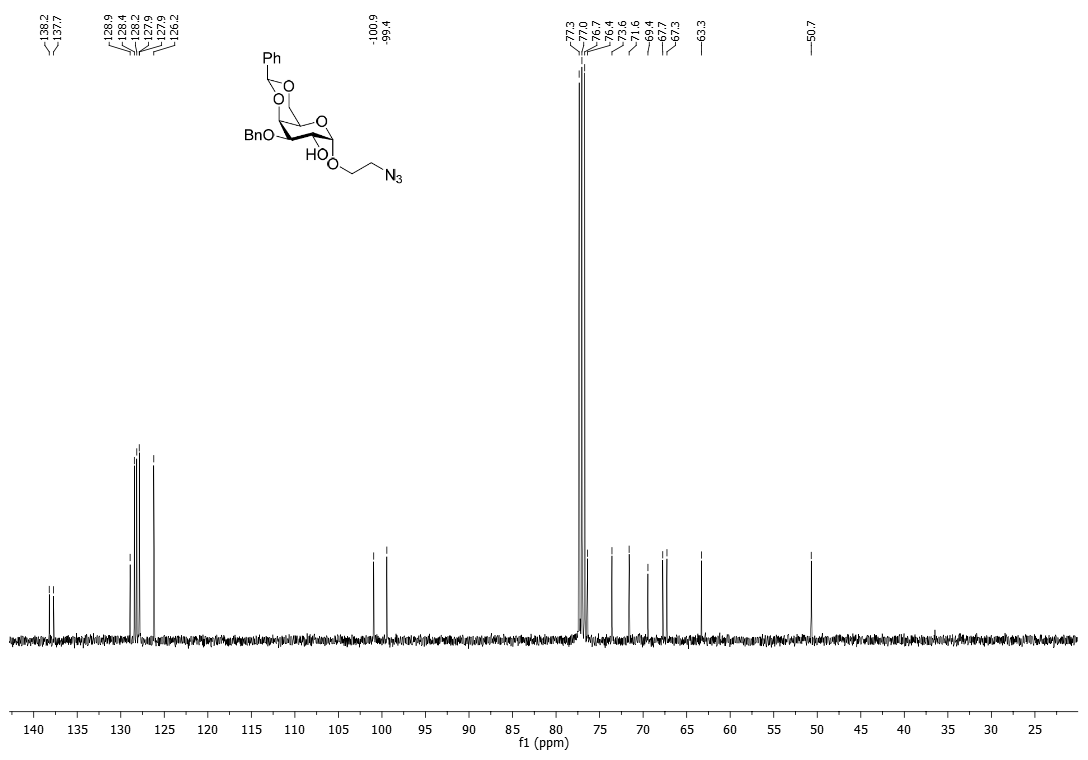


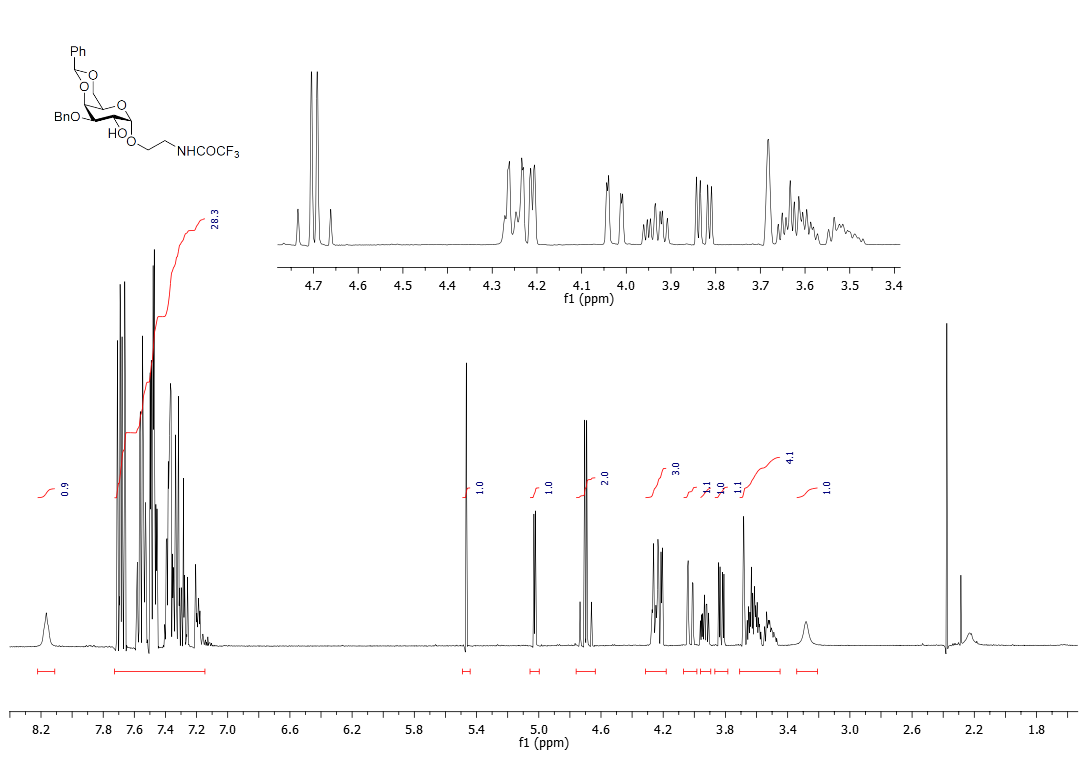


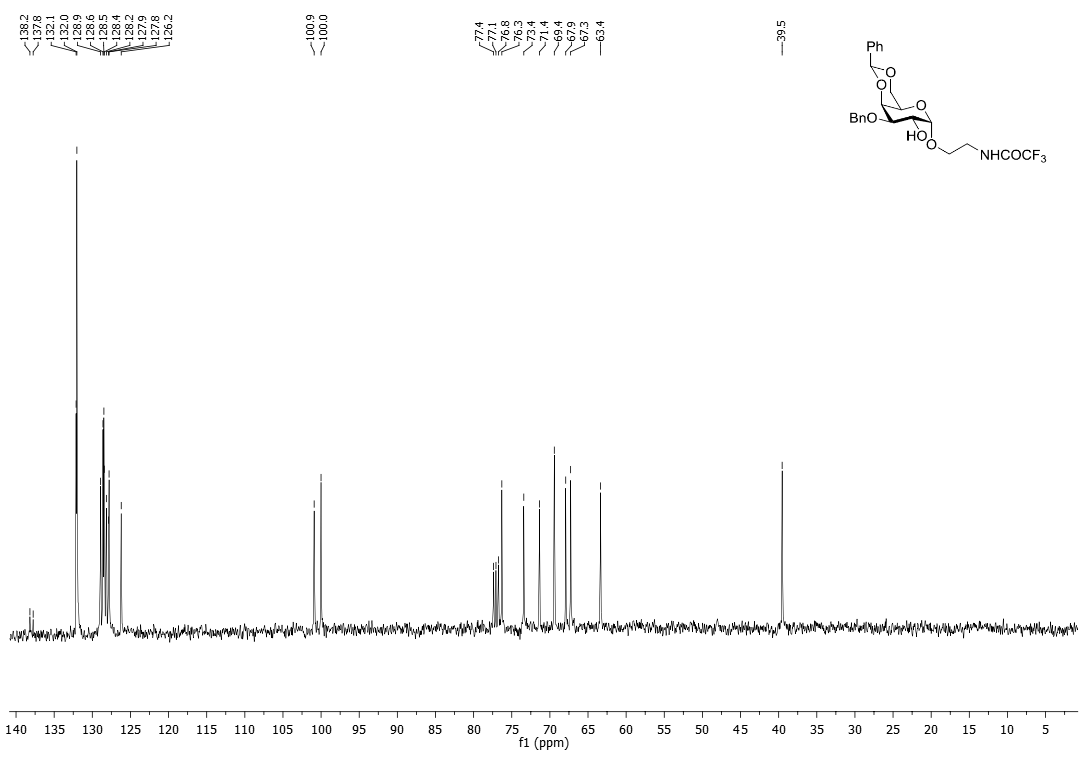


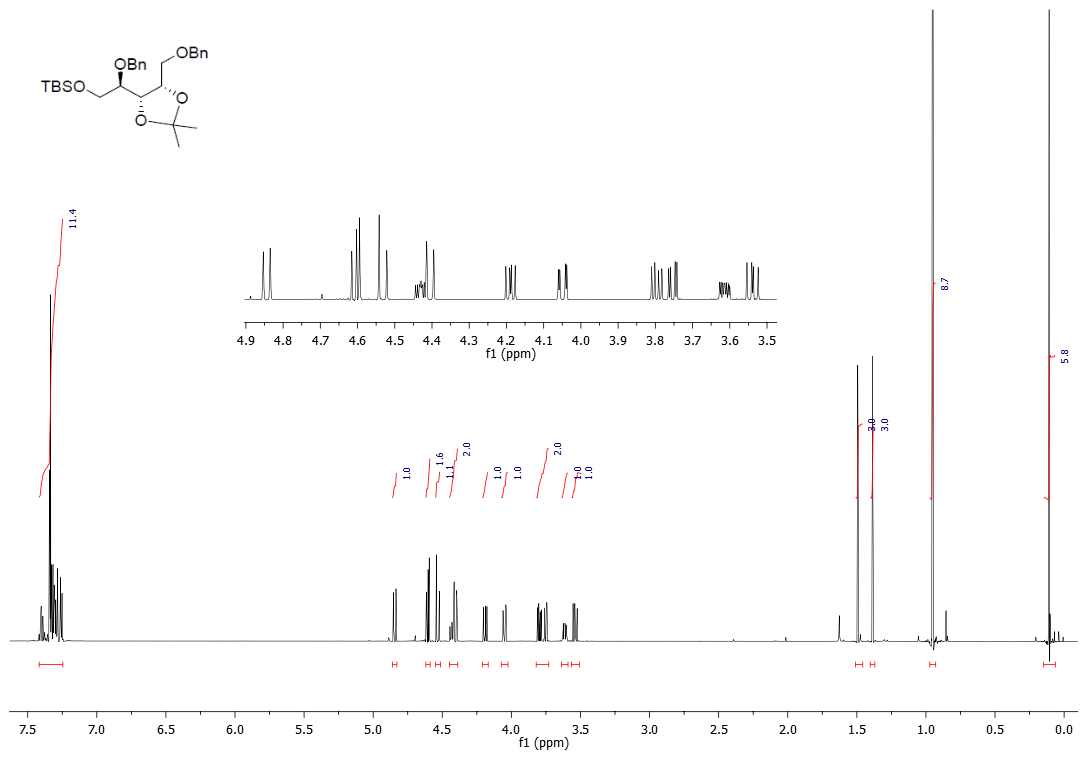


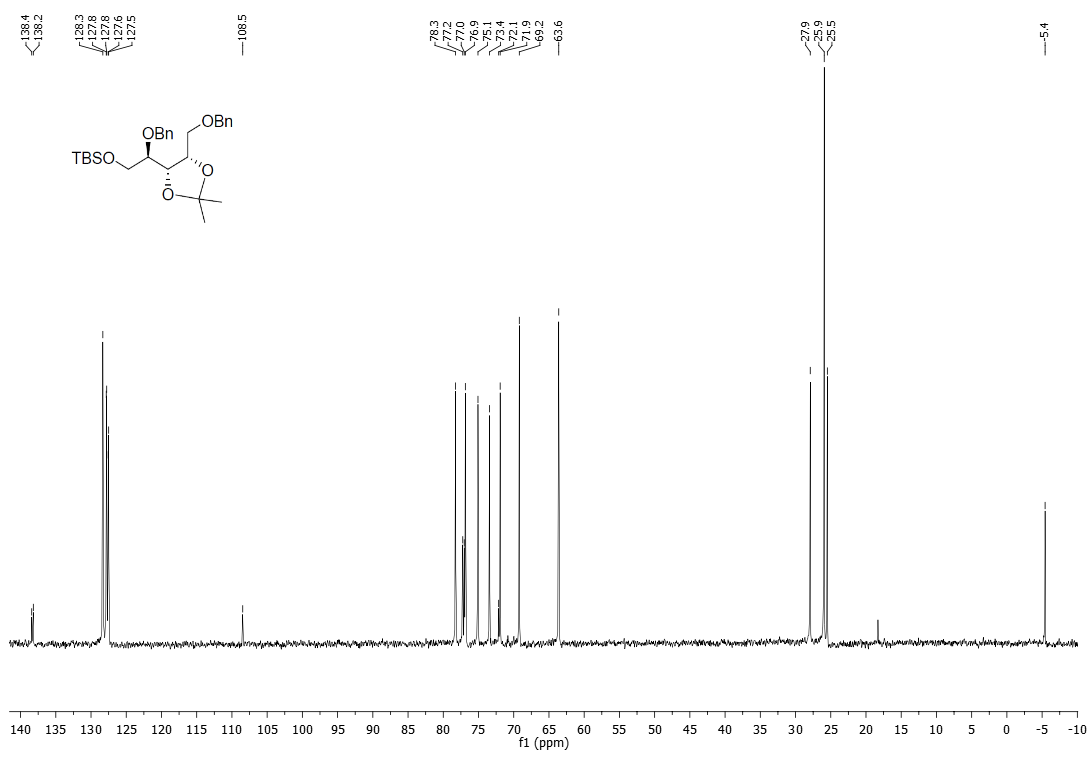


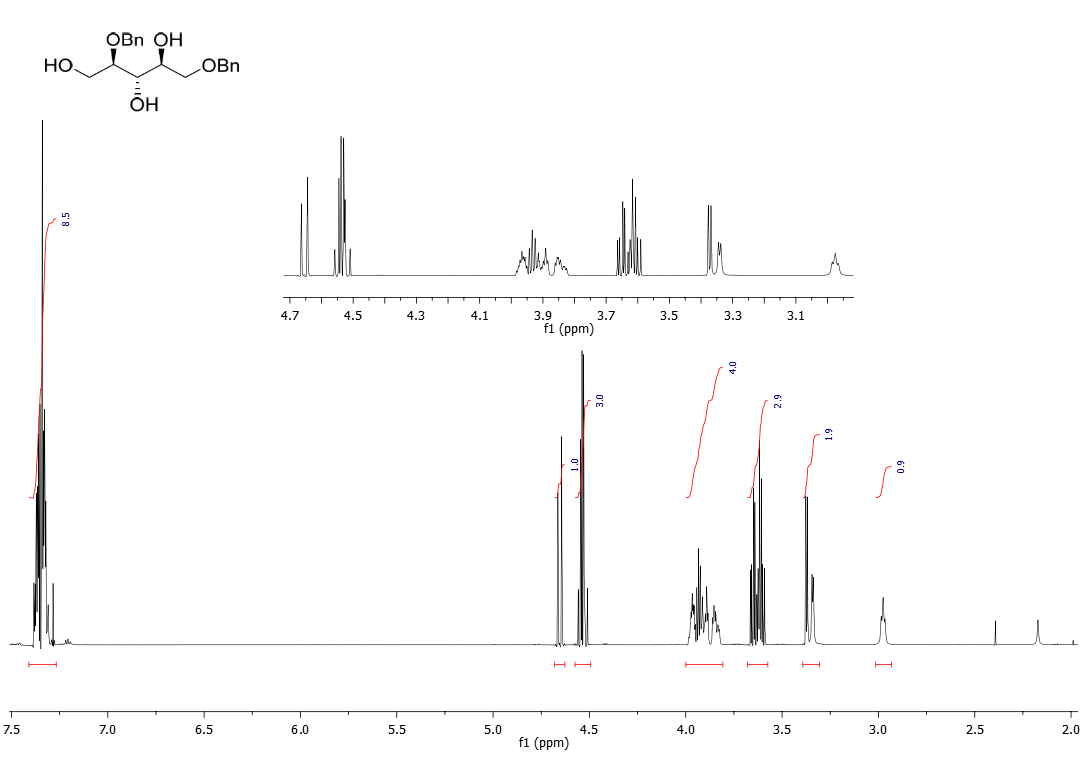


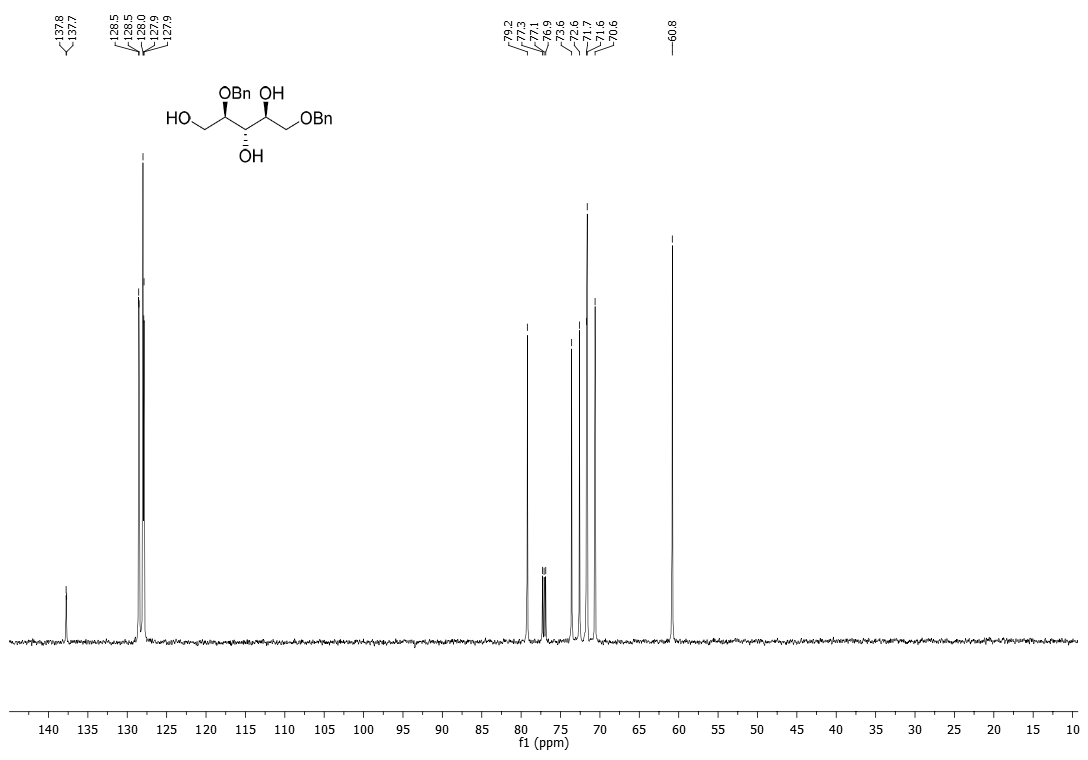


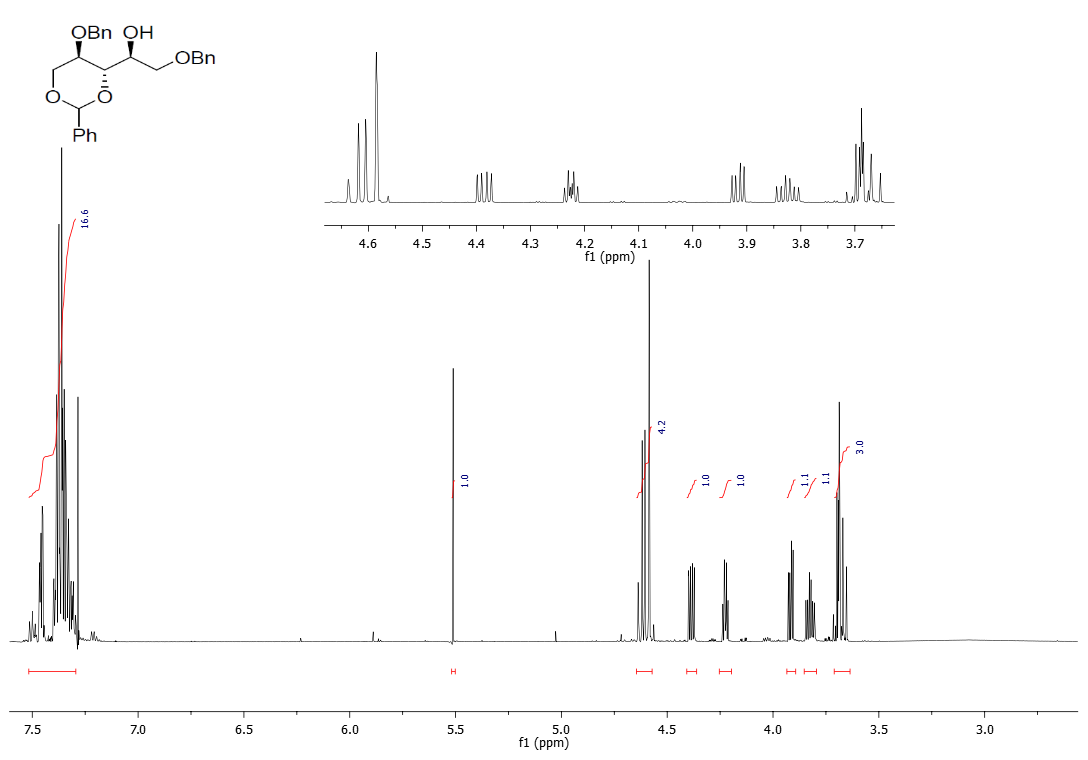


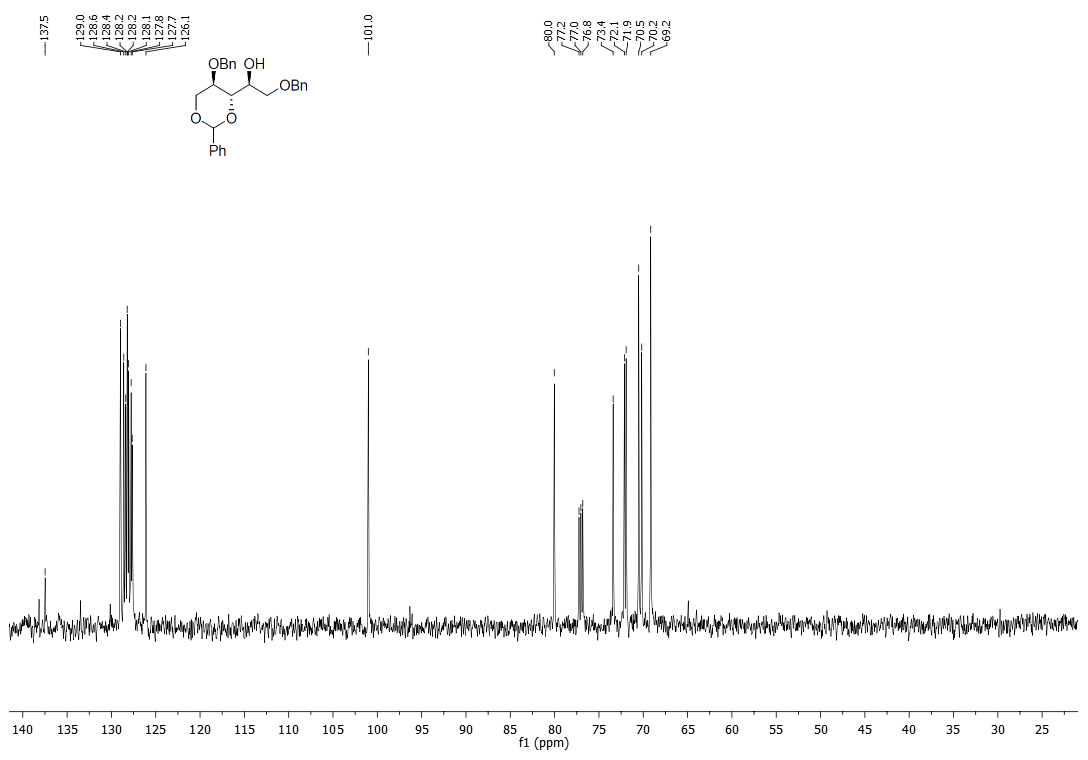


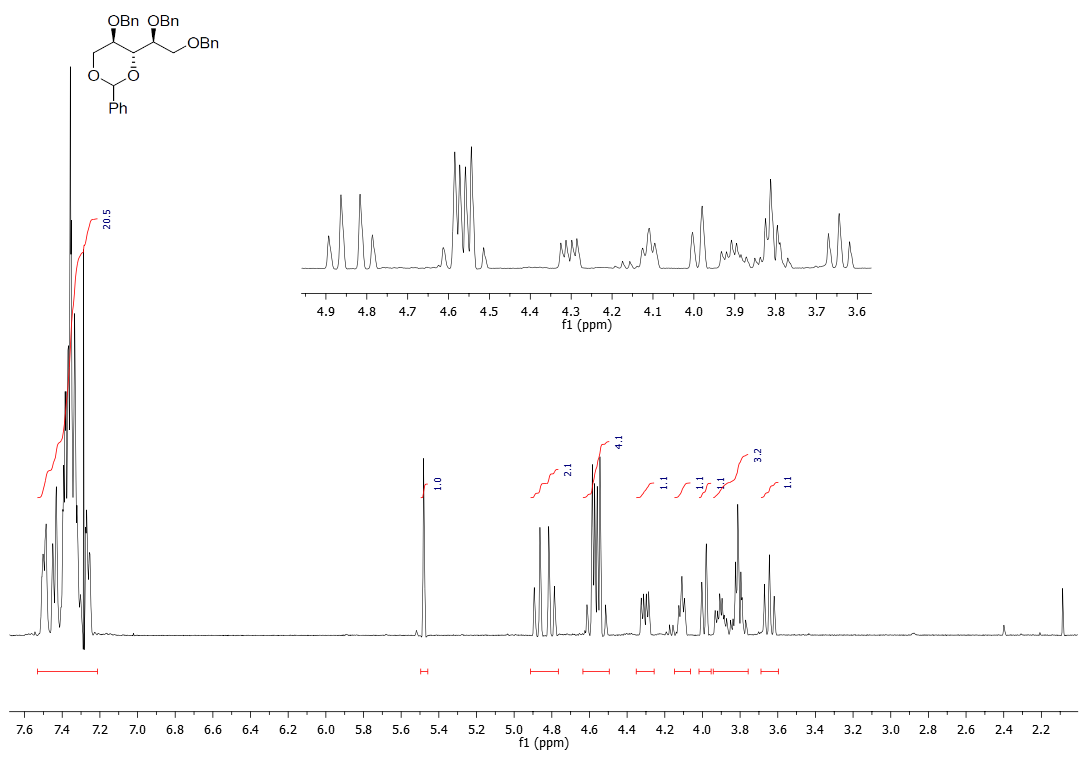


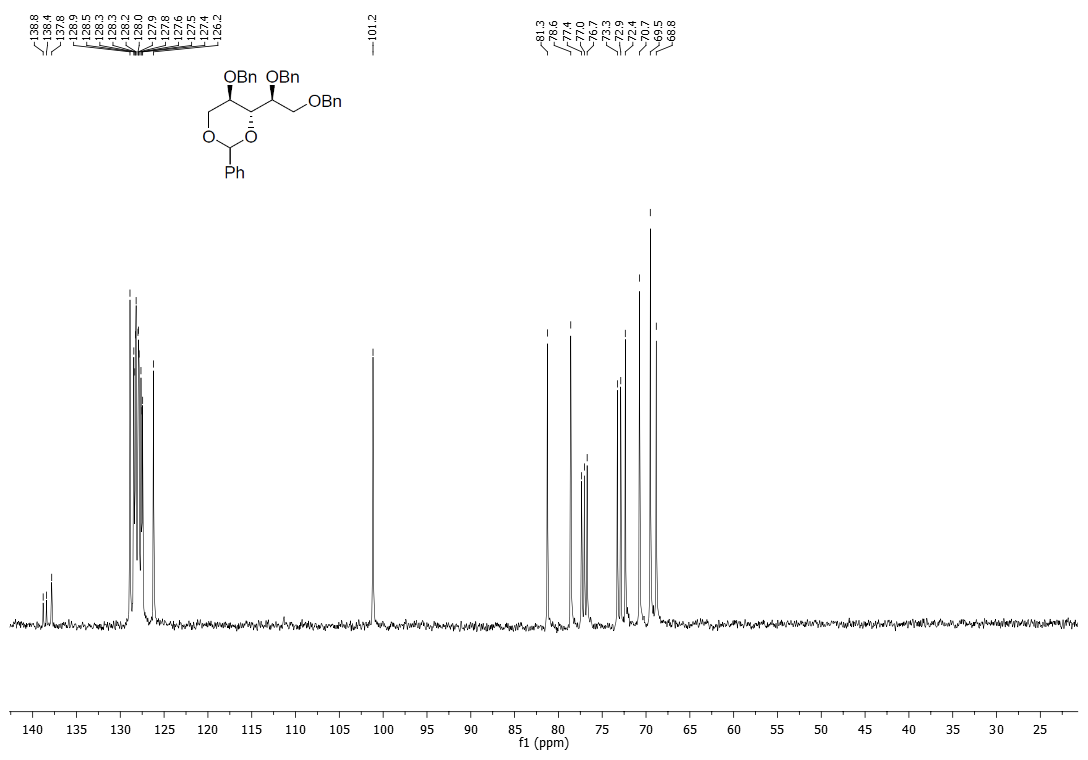


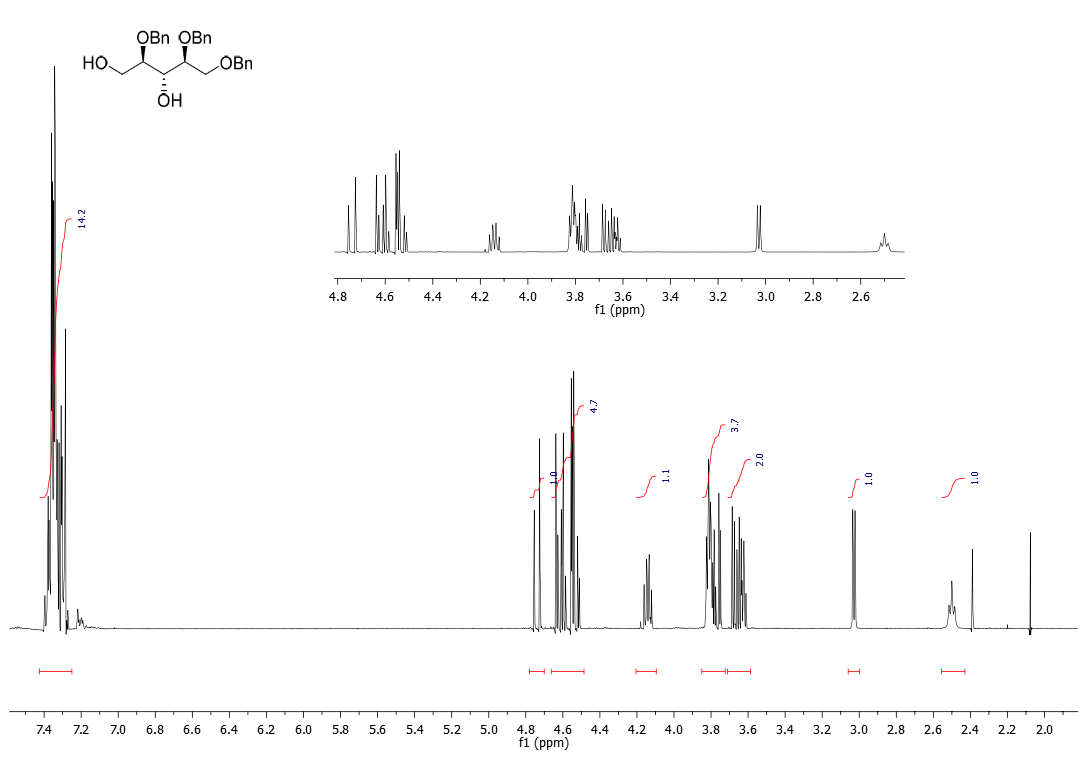


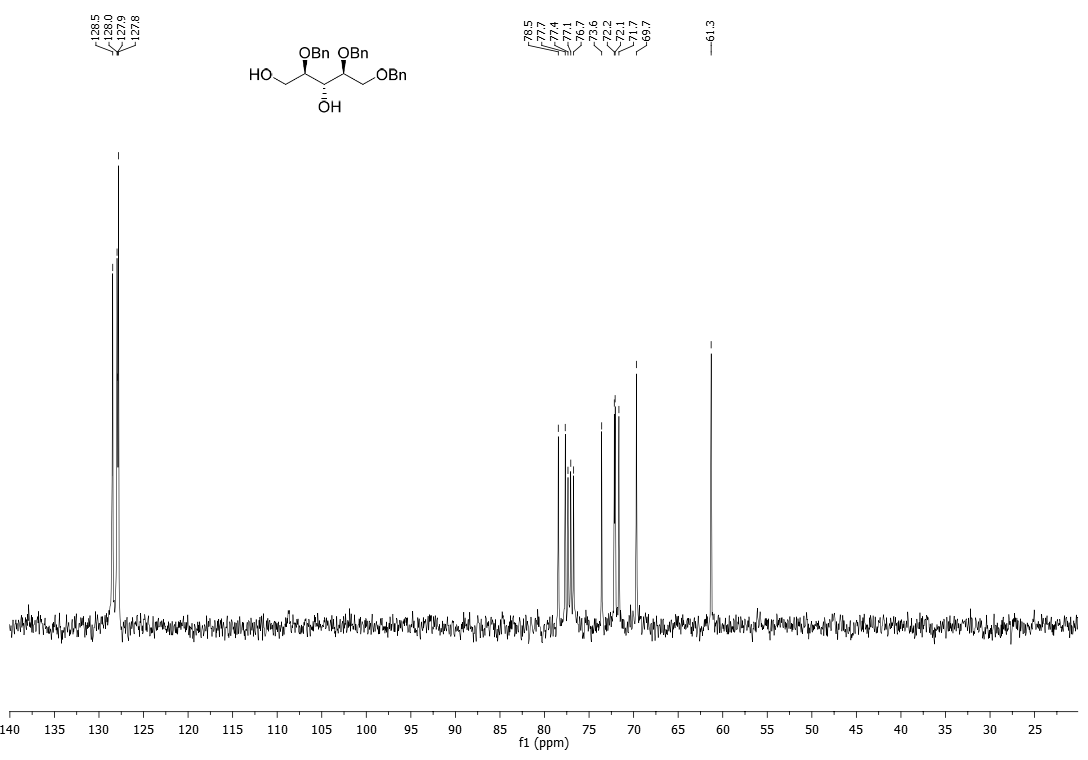


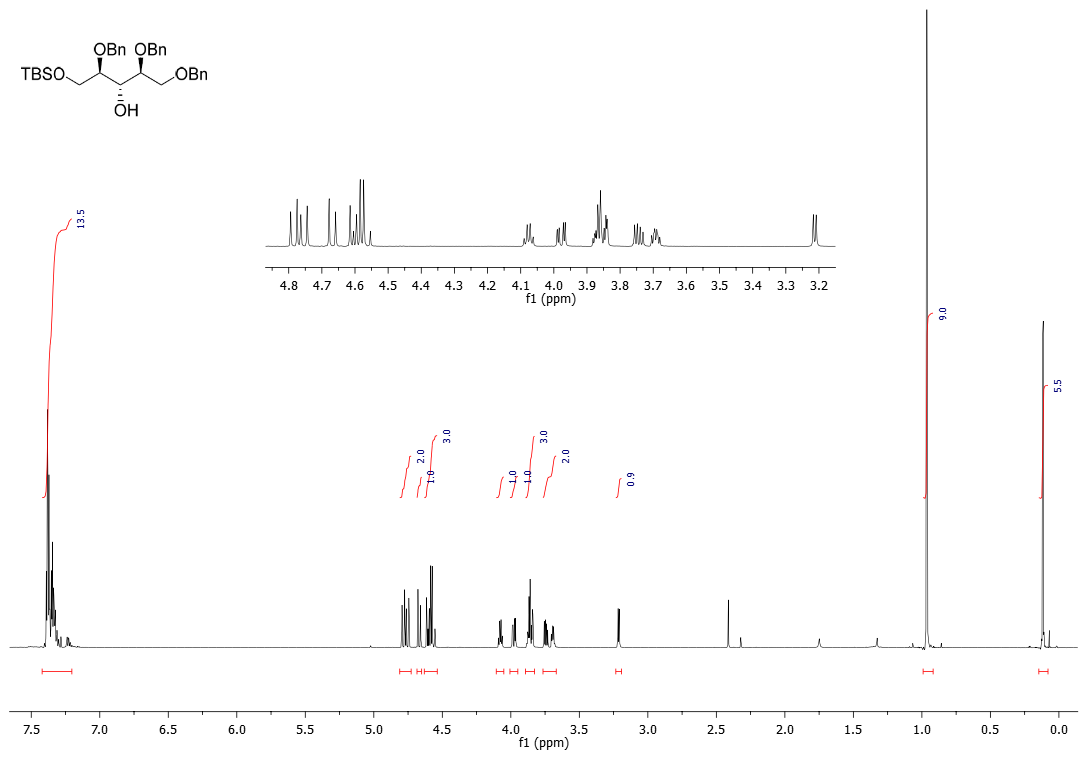


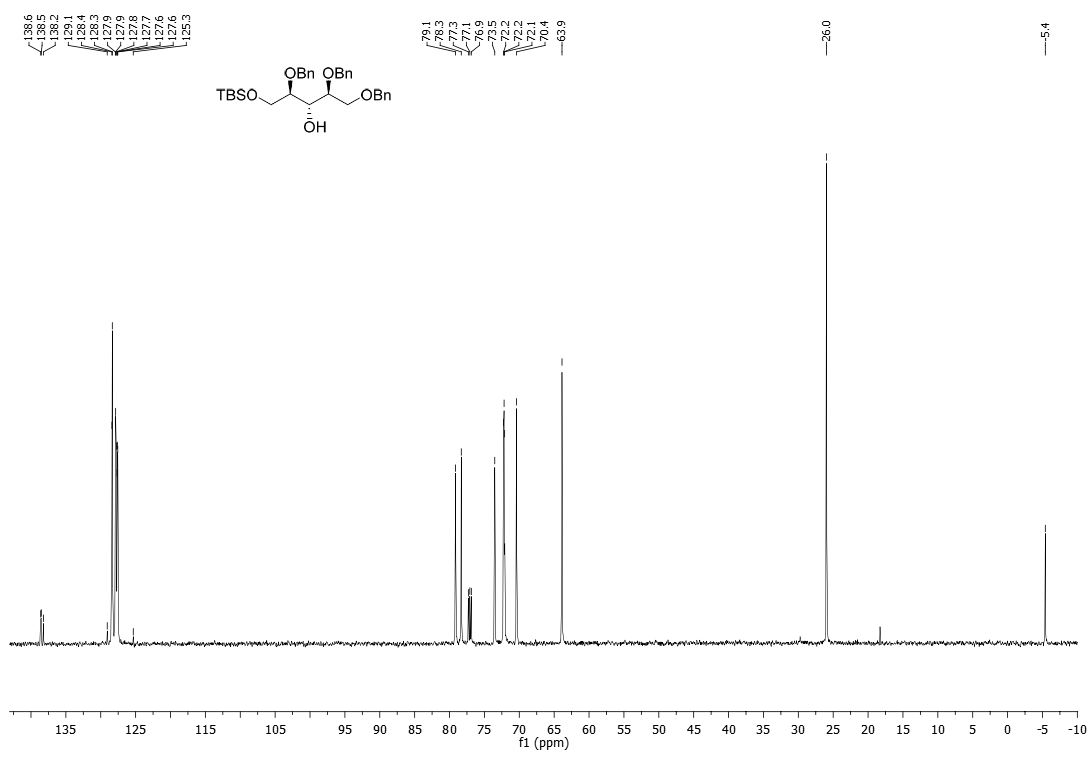


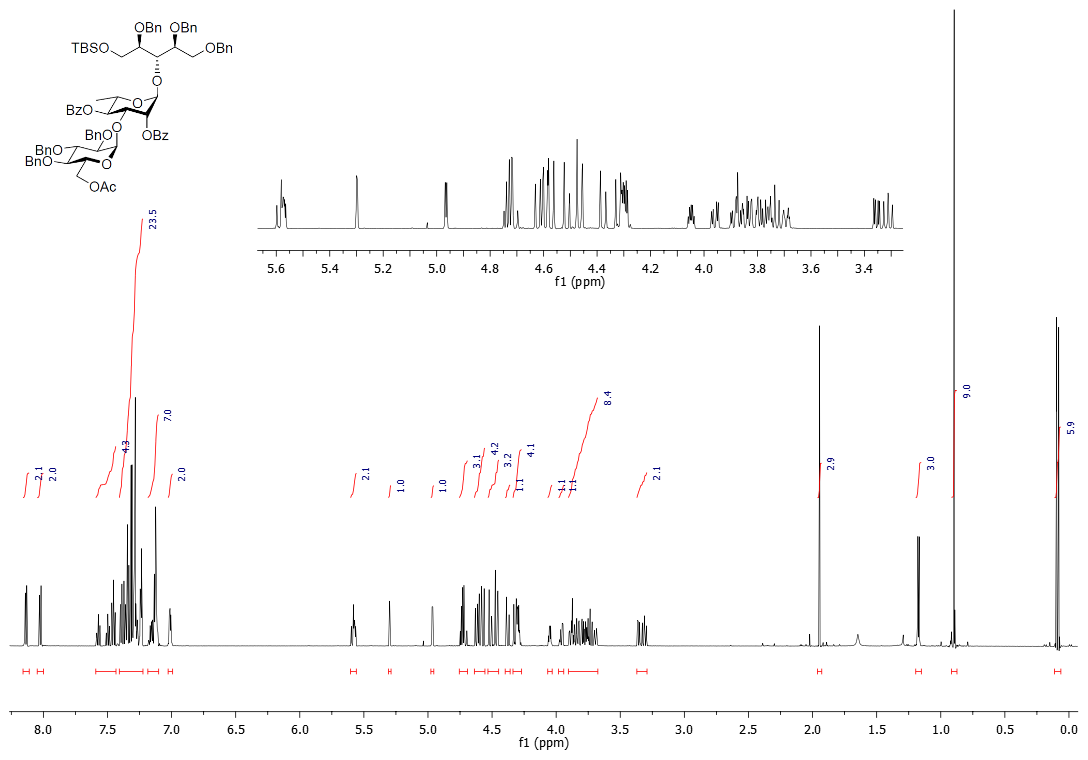


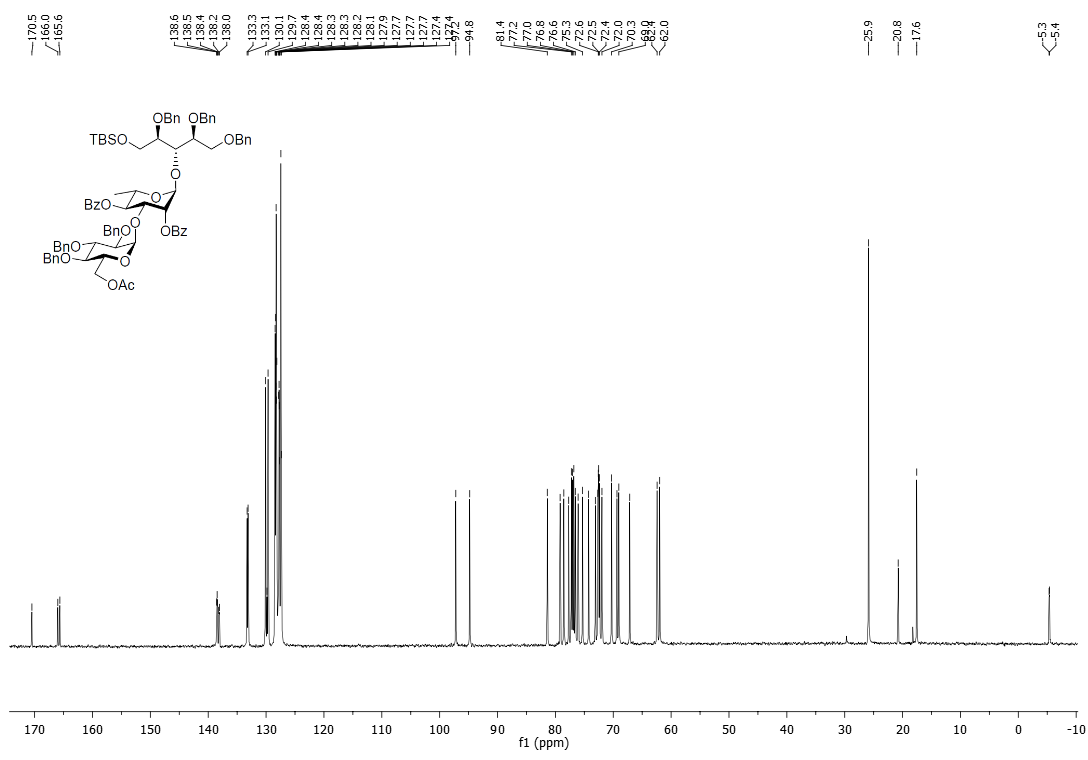


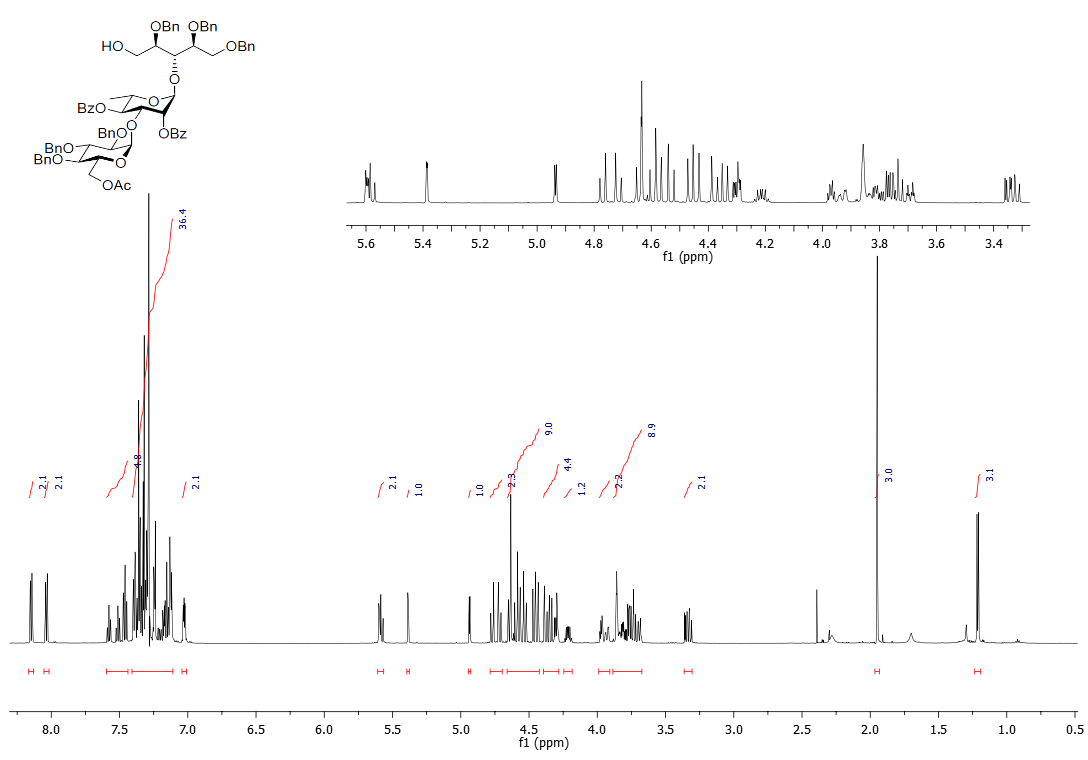


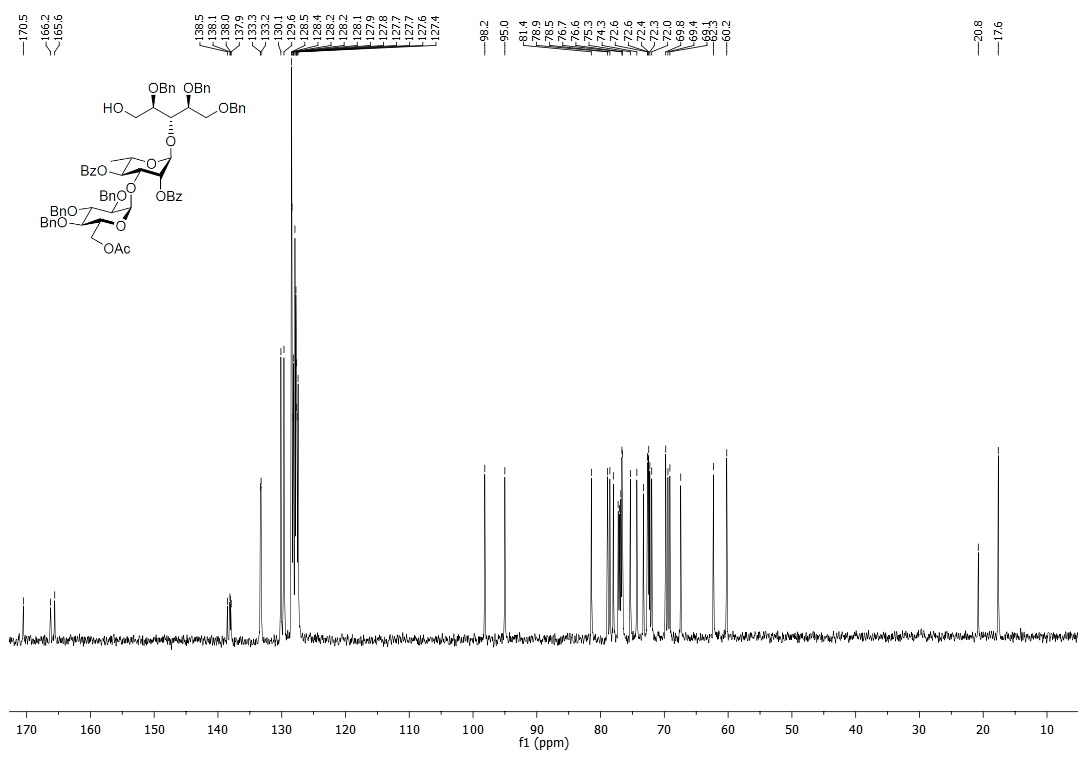


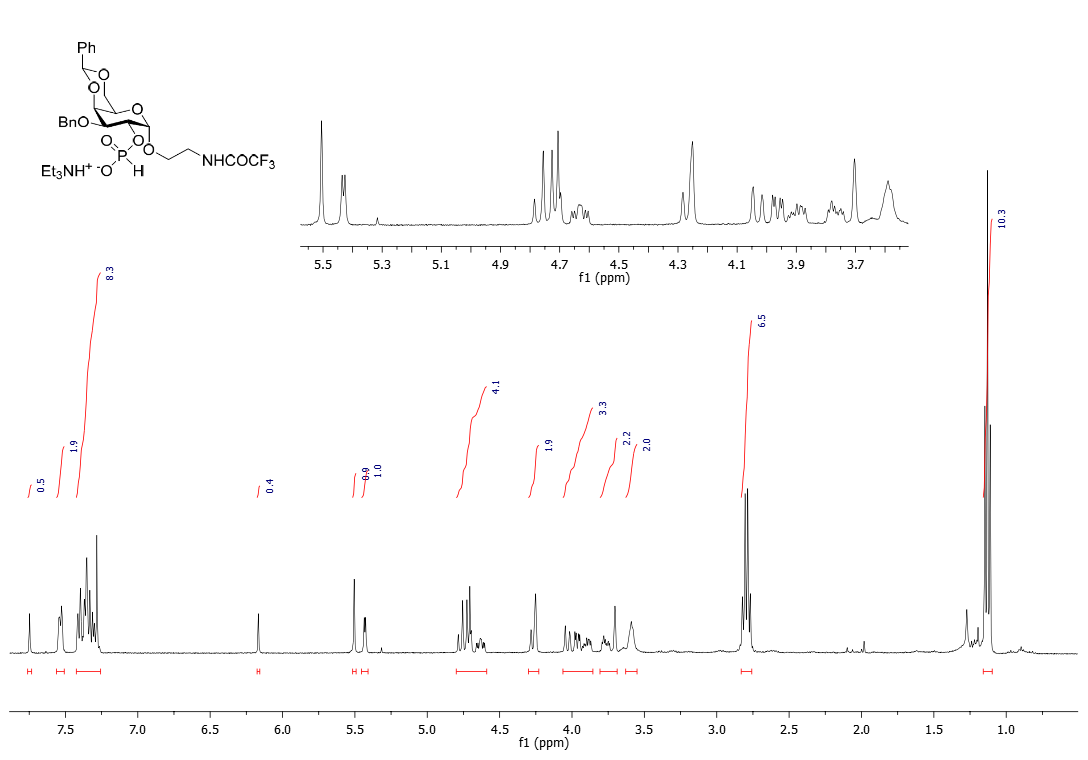


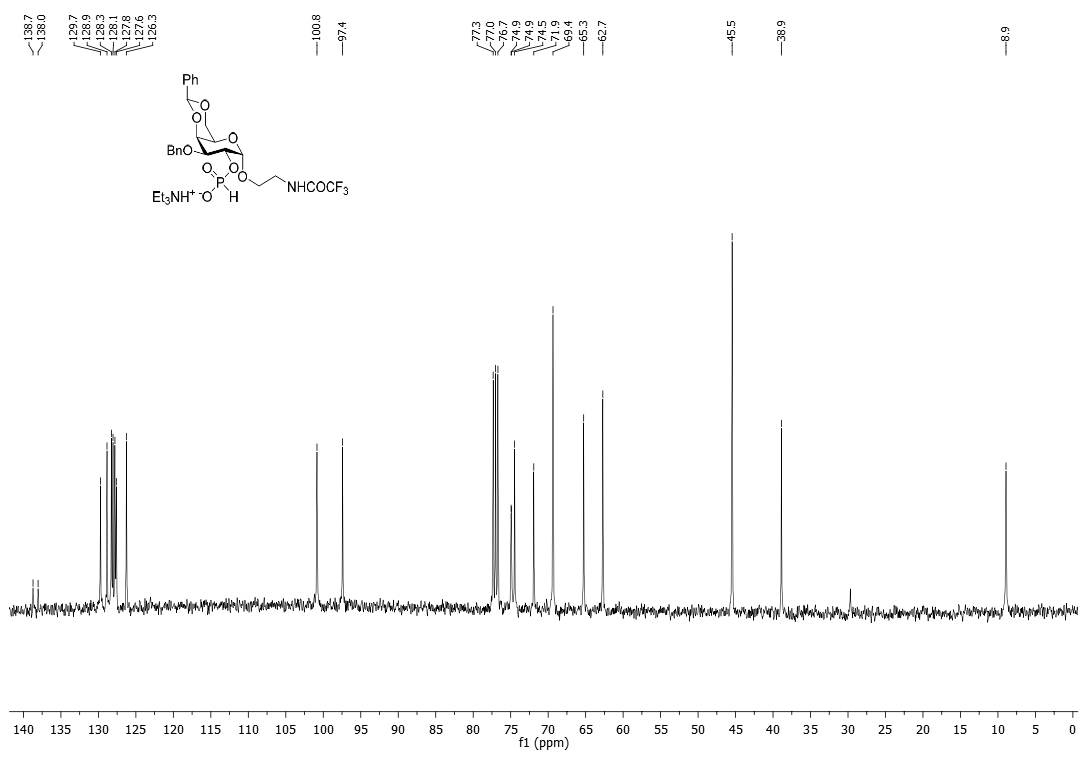


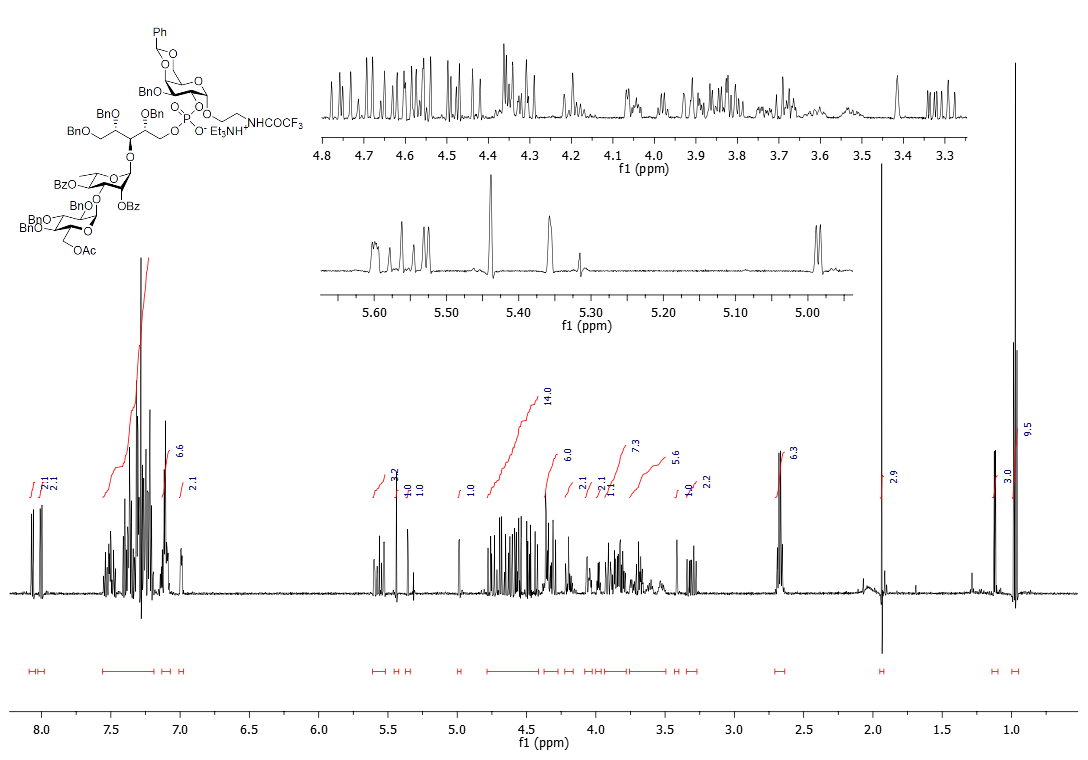


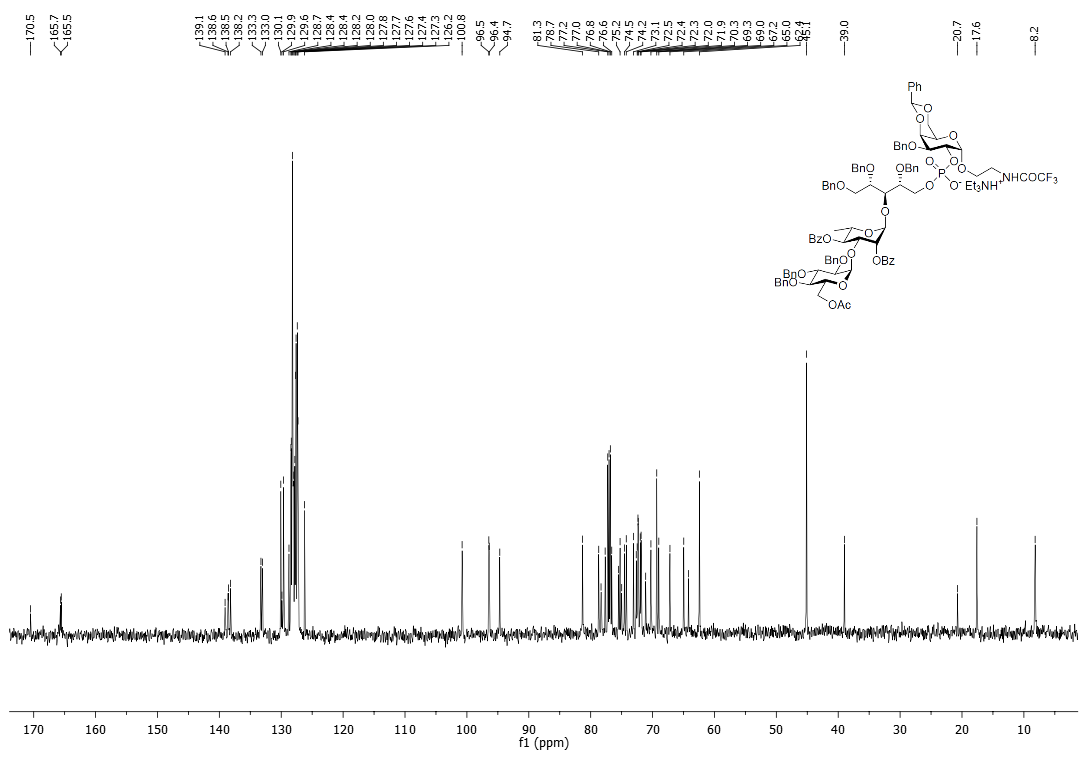


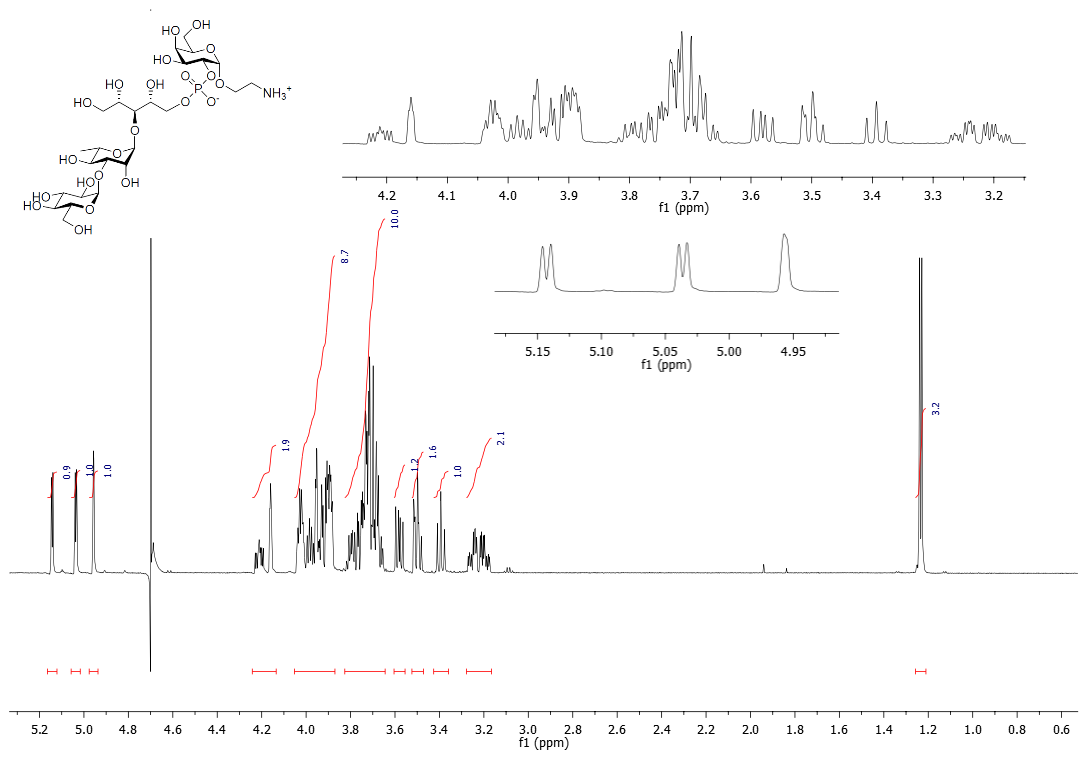


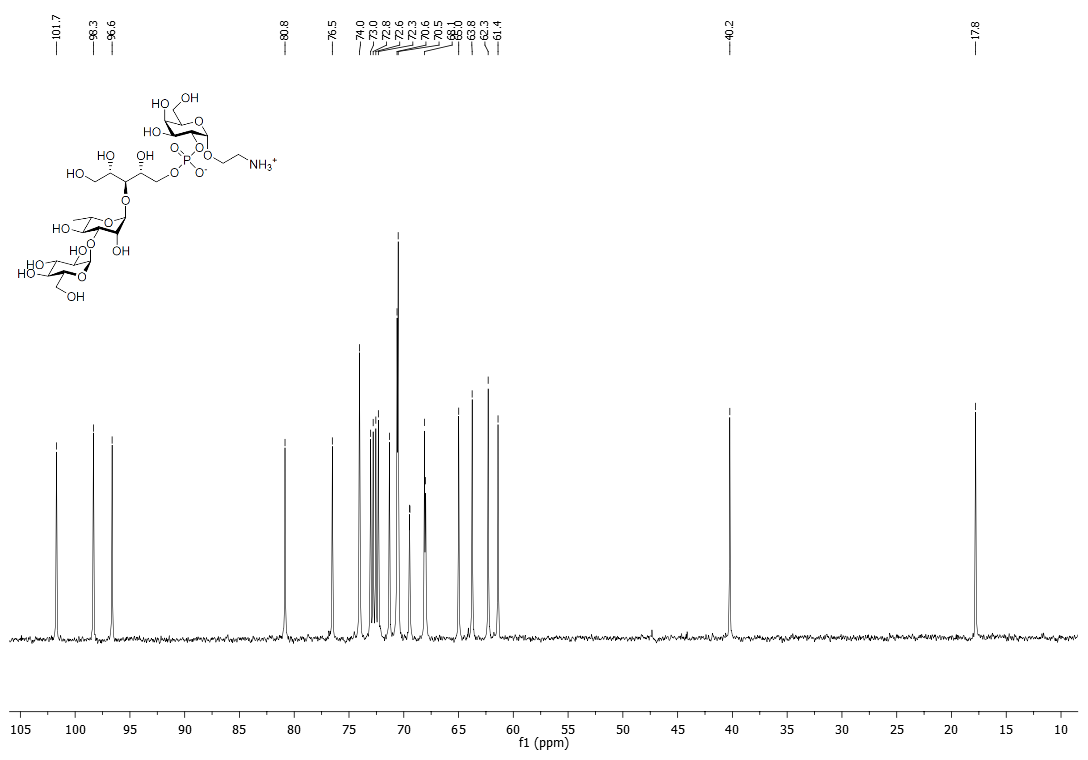


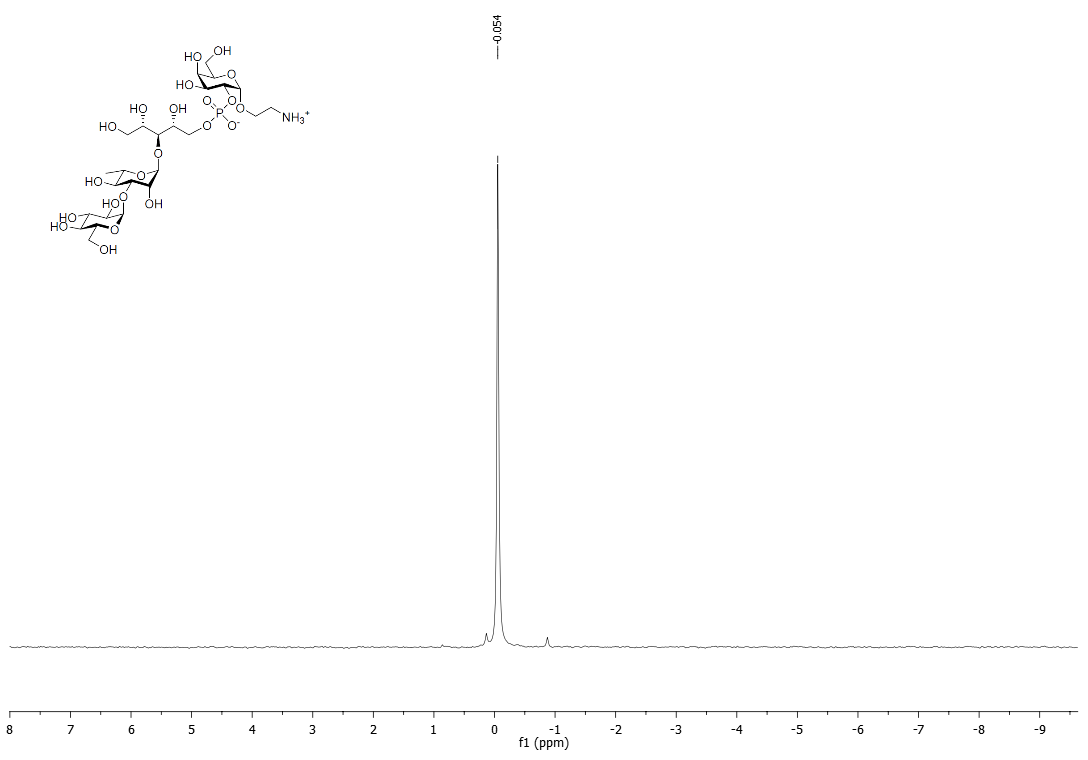


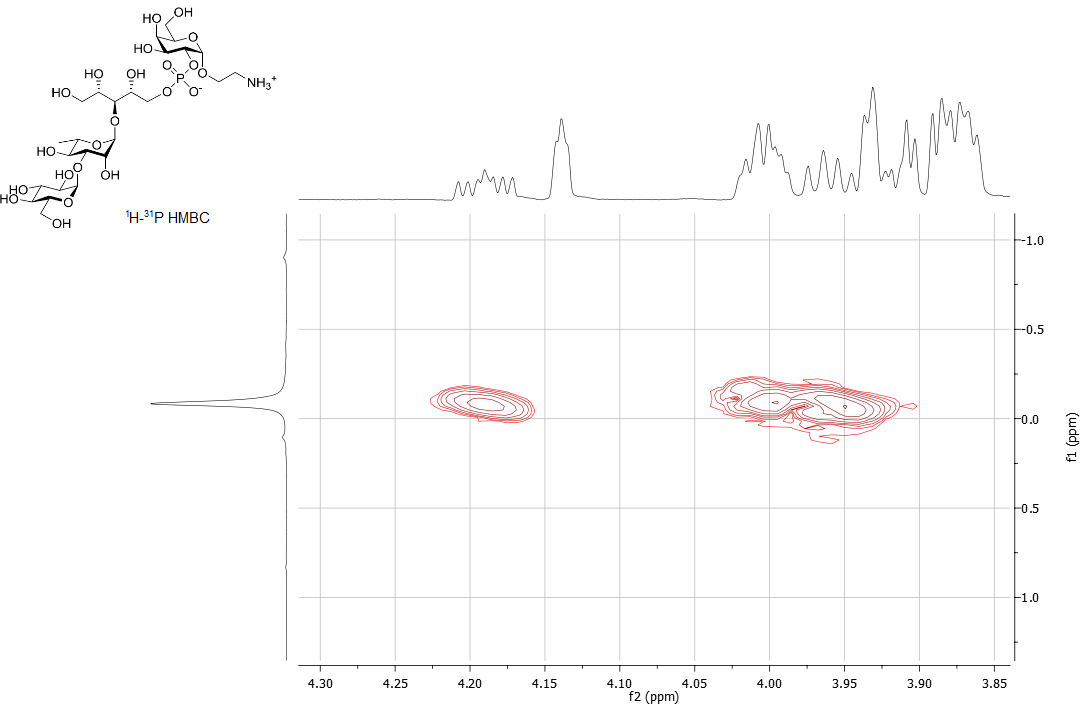


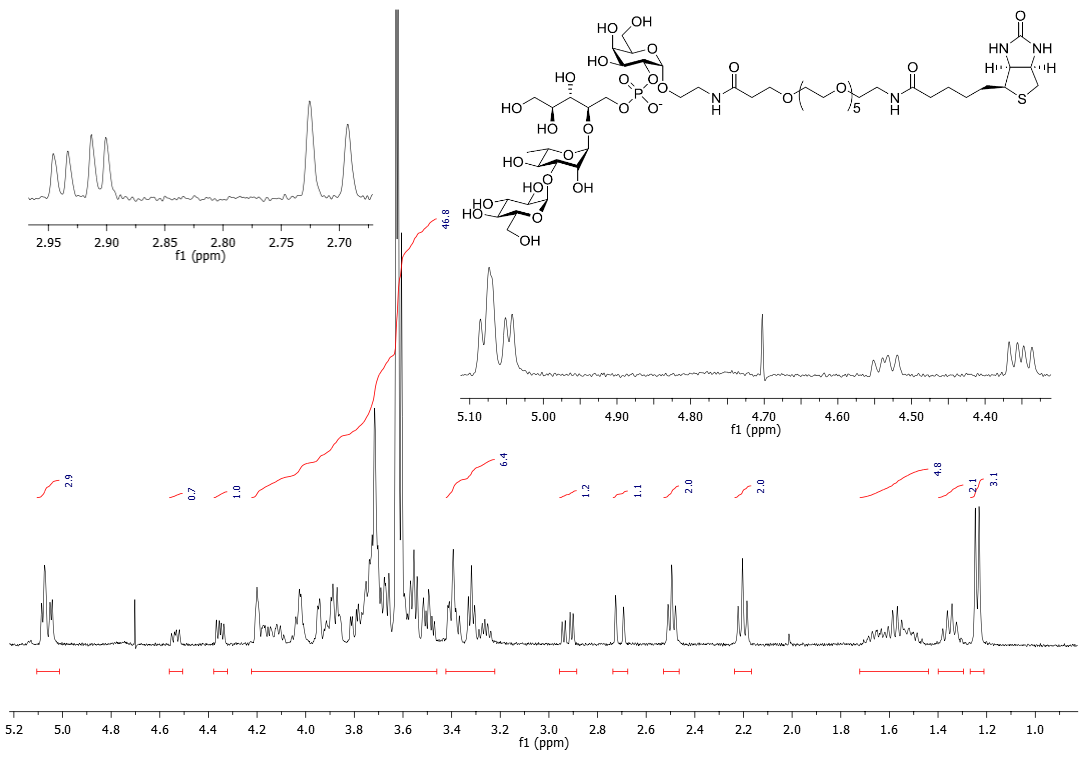


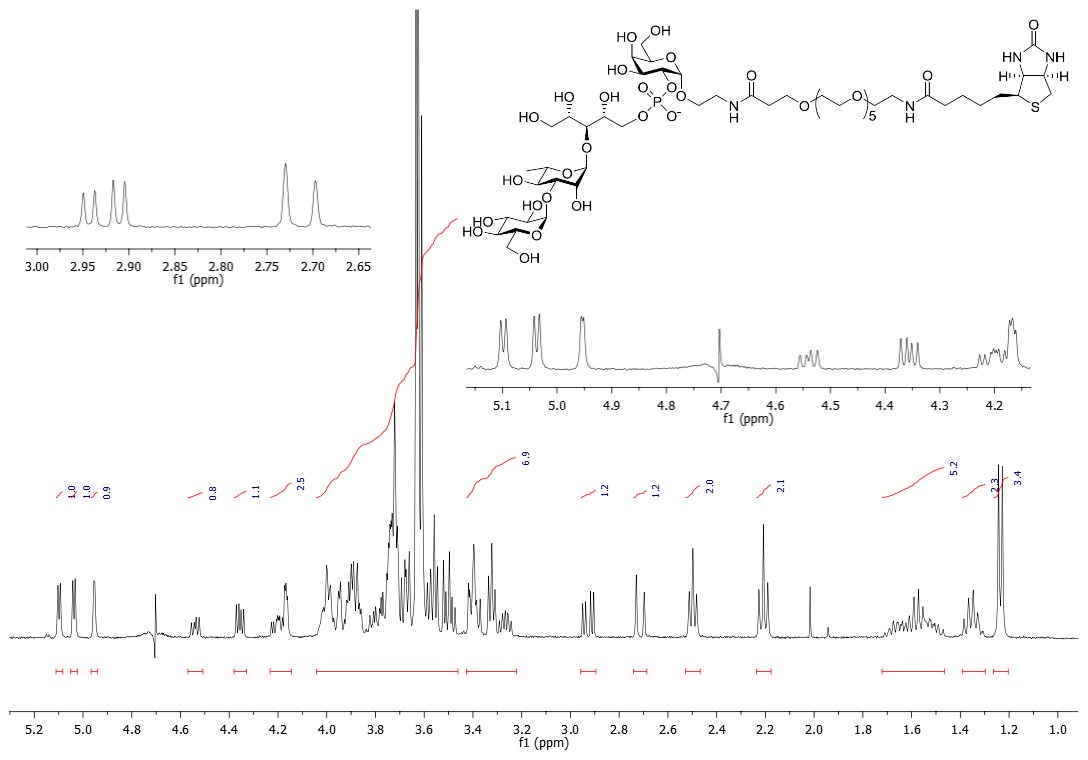

Supplement: Supplementary file 1 [file DataSheet1.docx]
